# Synthesis and Biological Evaluation of Novel Urea- and Guanidine-Based Derivatives for the Treatment of Obesity-Related Hepatic Steatosis

**DOI:** 10.3390/molecules19056163

**Published:** 2014-05-15

**Authors:** Xiaolin Liang, Heying Pei, Liang Ma, Yan Ran, Jinying Chen, Guangcheng Wang, Lijuan Chen

**Affiliations:** State Key Laboratory of Biotherapy, West China Hospital, West China Medical School, Sichuan University, Keyuan Road 4, Gaopeng Street, Chengdu 610041, China; E-Mails: liang_11@hsit.edu.cn (X.L.); pei_2014@hsit.edu.cn (H.P.); liang_m@scu.edu.cn (L.M.); ran_2014@hsit.edu.cn (Y.R.); chen_2011@hsit.edu.cn (J.C.); wang_gc12@hsit.edu.cn (G.W.)

**Keywords:** leptin, hepatic steatosis, urea and guanidine-based, diet-induced obesity

## Abstract

Leptin, the product of the obese gene, is an adipocyte-secreted protein hormone playing a key role in the progression of obesity and hepatic steatosis. In this study, 28 novel (thio)urea and guanidine-based analogues have been synthesized and N-(1-(4-(3-(2-chloroethyl)ureido)benzyl)piperidin-4-yl)-3-(trifluoromethyl) benzamide (**7i**) was found to be a potent regulator of leptin expression in 3T3-L1 adipocytes. Treatment with **7i** at a dose of 50 mg/kg/day for 35 days reduced the body weight and liver weight of diet-induced obesity mice by 13.5% and 18.4%, respectively, while also improving the serum levels of triglyceride, total cholesterol, leptin, adiponectin, LDL-c, HDL-c. Hematoxylin-eosin (H&E) and Oil Red O staining also confirmed that **7i** ameliorated fat deposition in liver tissue and restricted the size of adipocytes in obesity-related fatty liver disease.

## 1. Introduction

Obesity, with an increasing prevalence around the world, has been cited by the World Health Organization as one of the greatest public health challenges [1,2]. It is considered a serious health problem because of the high incidence of other metabolic syndromes such as type 2 diabetes, cardiovascular disease, and osteoarthritis seen in obese individuals [3,4]. In gastroenterology, obesity-related complications are also frequent, and the consequences of obesity for the liver have attracted more attention because of its crucial role in metabolism [5].

Hepatic steatosis, which manifests at a high rate in overweight or obese individuals as fat infiltration and deposition, is a precursor of advanced non-alcoholic fatty liver diseases (NAFLDs) [6,7]. Currently, 34% of the general population and over 75% of obese individuals are estimated to have fatty liver diseases [8,9,10]. The high association between obesity and fatty liver disease may result from the serum concentration changes of several important adipokines and one such adipokine is the 16 kDa protein hormone leptin [11]. Leptin, a cytokine secreted by adipose tissue, plays a central role in the regulation of energy balance via the activation of leptin receptors, particularly within the central nervous system [12]. It has been demonstrated that the serum leptin concentration in obese subjects is higher than in non-obese subjects, with a significant correlation between body weight and circulating serum leptin levels [13,14].

Although the actual mechanisms leading to hepatic steatosis in obese individuals remain unclear, insulin resistance (IR) and abnormal lipid metabolism are generally accepted as the essential risk factors of obesity-related fatty liver disease [15]. In fact, elevated leptin levels in obesity inhibit insulin signaling and enhance the IR effect, which leads to an increase in intracellular fatty acids and deposition of triglycerides (TG) in the hepatocytes [16,17,18,19,20]. Recent studies also showed that obesity-induced leptin functions directly in promoting the progression of non-alcoholic steatohepatitis [21,22,23]. These compelling evidences suggested that leptin could be a potential therapeutic target of obesity-related fatty liver disease. 

**Figure 1:**
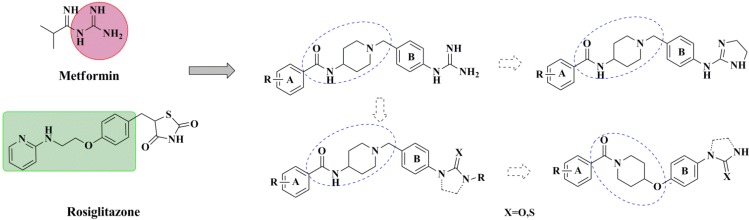
Rationale for the design of compounds **7a–l**, **8a–c**, **14a–d**, **17a–i**.

As insulin sensitizers, metformin and rosiglitazone have been proved effective in the clinic to reduce the serum leptin levels and improve the situation of hepatic steatosis [24,25,26]. However, it was generally accepted that the use of TZDs would cause hepatotoxicity and increase the risk of heart failure [27,28], which has provided the impetus to develop a series of novel compounds integrating the structural characteristics of metformin and rosiglitazone and investigate their pharmaceutical functions against obesity-related fatty liver disease. As shown in Figure 1, the long chain motif of rosiglitazone has been kept with the changed 4-formamide-*N*-methylpiperidine or 4-hydroxy-*N*-ethanonepiperidine bridges between two benzene rings, and then guanidine was introduced to the *par*a-position of ring B where a substituted thiazolidine was originally. Furthermore, as guanidine’s isosteric urea core has also shown capacity in decreasing liver TG [29], this encouraged us to try to replace guanidine with (thio)urea moieties. As a result compound 7i was discovered to be a good leptin regulator and to improve hepatic steatosis symptoms in DIO mice.

## 2. Results and Discussion

### 2.1. Chemistry

**Scheme 1:**
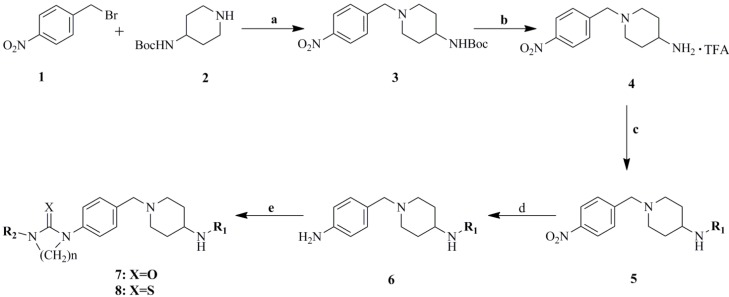
Synthesis of compounds **7a–l** and **8a–c**.

These urea derivatives **7** and thiourea derivatives **8** were prepared using the protocol outlined in Scheme 1. Compound **3** was synthesized via the reaction of 4-nitrobenzyl bromide and *tert*-butyl piperidin-4-ylcarbamate in the presence of K_2_CO_3_ as base and acetone as solvent. Then, the *tert-*butyloxycarbonyl (Boc) protection was removed in TFA at 0 °C and treatment of the resulting trifluoroacetate with various substituted benzoic acids in the presence of EDCI and DMAP yielded compounds **5**. The chemical reduction was conducted in anhydrous EtOH under reflux in the presence of SnCl_2_ to give the corresponding amines **6**. Finally, treatment of various amines **6** with the corresponding iso(thio)cyanates in DCM at 0 °C afforded the desired (thio)urea derivatives **7** and **8** (Scheme 1 and Table 1). 

*Reagents and conditions*: (a) K_2_CO_3_, acetone, 70 °C, 4 h, 95.2%; (b) TFA, DCM, 0 °C to room temperature, 3 h, 96.8%; (c) R_1_COOH, EDCI, DMAP, DCM, room temperature, overnight, 65%–75%; (d) SnCl_2_, EtOH, 2 h, 80 °C, 45%–75%; (e) method A for **7a–7i**, **8**: substituted iso(thio)cyanate, DCM, 0 °C to room temperature, 30%–70%; method B for **7j–7l**: NaH, correspond **7**, THF, 0 °C to room temperature, 30%–70%.

**Table 1:** Structural and Inhibition Effects of Leptin Expression on 3T3-L1 of **7a-l**, **8a-c**. 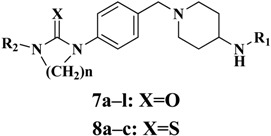

No.	n	R_1_	R_2_	Inhibition of Leptin expression in 3T3-L1 (%) ^a^
**7a**	0	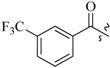	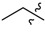	84.3
**7b**	0	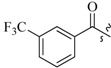	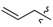	67.4
**7c**	0	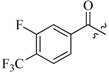	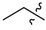	67.5
**7d**	0	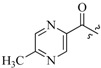	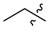	54.3
**7e**	0	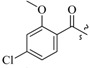	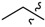	73.9
**7f**	0	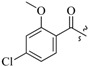	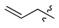	68.1
**7g**	0	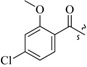	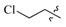	77.1
**7h**	0	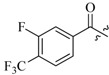	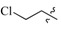	74.5
**7i**	0	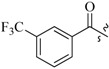	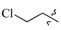	89.0
**7j**	2	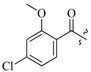	H	54.1
**7k**	2	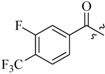	H	69.2
**7l**	2	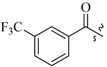	H	78.4
**8a**	0	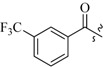	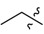	61.4
**8b**	0	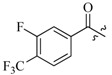	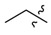	48.1
**8c**	0	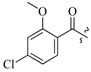	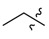	45.0
**Metformin**				39.3

^a^ the result of inhibition effect were results were recorded from three independent experiments.

**Scheme 2:**
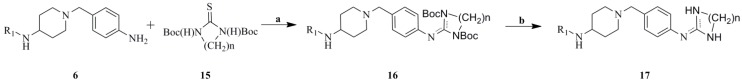
Synthesis of compounds **17a–i**.

Intermediates **6** were treated with *N,N'*-di(*tert*-butoxycarbonyl)thiourea in the presence of triethylamine (TEA) and mercury (II) chloride to give **16**, as described in Scheme 2. Deprotection of **16** yielded the desired guanidine analogues **17** (Scheme 2 and Table 2).

*Reagents and conditions*: (a) *N*,*N'*-Bis-*tert*-butoxycarbonylthiourea(or imidazolidinethione), HgCl_2_, TEA, EtOH, DCM, 0 °C to room temperature, overnight, 30%–50%; (b) TFA, DCM, 0 °C to room temperature, 3 h, 20%–40%.

**Table 2:** Structural and Inhibition Effects of Leptin Expression on 3T3-L1 of **17a–i**. 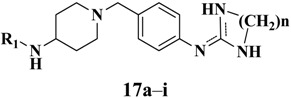

No.	n	R_1_	Inhibition of Leptin expression in 3T3-L1(%) ^a^
**17a**	**0**	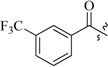	53.0
**17b**	**0**	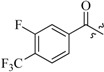	42.5
**17c**	**0**	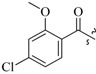	42.3
**17d**	**0**	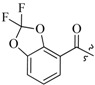	22.8
**17e**	**0**	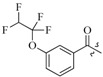	15.1
**17f**	**0**	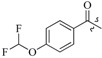	27.5
**17g**	**0**	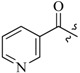	34.5
**17h**	**2**	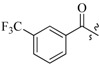	33.6
**17i**	**2**	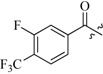	18.7
**Metformin**			39.3

^a^ the result of inhibition effect were results were recorded from three independent experiments.

**Scheme 3:**
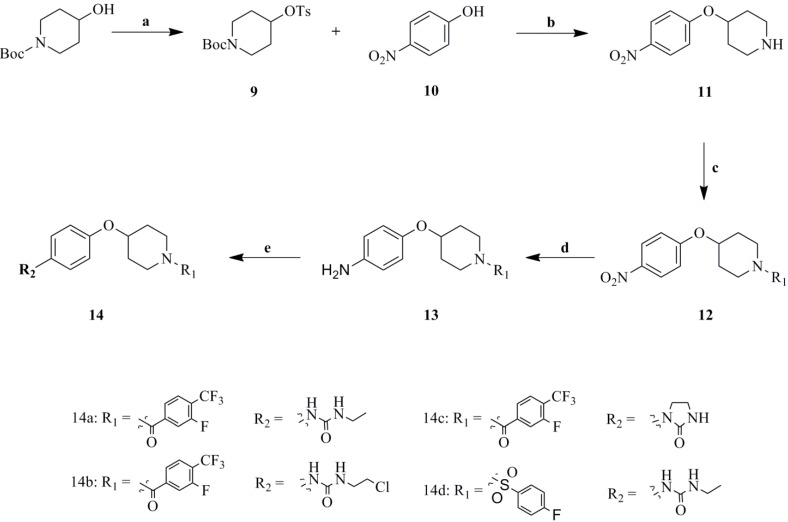
Synthesis of compounds **17a–i**.

Then, to explore the structure activity relationship (SAR) further, 4-piperidinol-substituted urea analogues have been synthesized as described in Scheme 3. 

*Reagents and conditions*: (a) TsCl, pyridine, 0 °C to room temperature, overnight, 95.2%; (b) (i) K_2_CO_3_, DMF, 100 °C, 89.7%, 8 h; (ii) TFA, DCM, 0 °C to room temperature, 3 h, 97.1%; (c) R_1_COOH, EDCI, DMAP, DCM, room temperature, overnight, 65%–75%; (d) Pd/C, HCOONH_4_, MeOH, 2 h, 70 °C, 45%–75%; (e) method A for **14a**, **14b**, **14d**: substituted iso(thio)cyanate, DCM, 0 °C to room temperature, 30%–70%; method B for **14c**: NaH, **14b**, THF, 0 °C to room temperature, 30%–70%.

The preparation of 4-piperidinol-substituted urea analogues was carried out via treatment of *tert*-butyl 4-hydroxypiperidine-1-carboxylate with *p*-toluenesulfonyl chloride (TsCl) at 0 °C in pyridine as solvent giving **9** in a good yield. The reaction of **9** and 4-nitrophenol using K_2_CO_3_ as a base, and subsequent deprotection of the Boc group yielded **11**. The pivotal amines **13** were obtained via the Pd/C reduction of **12** in MeOH at reflux. Compounds **14** were obtained by the same methods as **7** and **8** (Scheme 3 and Table 3).

**Table 3:** Structural and Inhibition Effects of Leptin Expression on 3T3-L1 of **14a–c**. 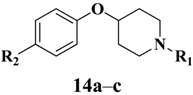

No.	R_1_	R_2_	Inhibition of Leptin expression in 3T3-L1 (%) ^a^
**14a**	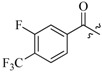	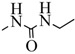	42.5
**14b**	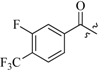	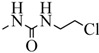	45.0
**14c**	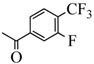	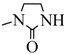	50.3
**14d**	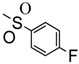	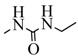	24.8
**Metformin**			39.3

^a^ The result of inhibition effect were results were recorded from three independent experiments.

### 2.2. Expression of Leptin in 3T3-L1 Adipocytes

During the *in vitro* screening, these compounds containing (thio)urea and guanidine were evaluated at 10 μM for their activity in decreasing leptin expression in 3T3-L1 adipocytes. Metformin was used as positive control and all tests were performed three times using each agent (Table 1, Table 2 and Table 3).

As shown in Table 1, leptin expressions was all inhibited after treatment with (thio)urea analogues at 10 μM. Compound **7i** exhibited the most significant activity and reduced the leptin level by 89.0% compared to metformin which decreased leptin by 39.3%. Further inspection of their structural features showed that the potencies of the thiourea analogues 8 were in general inferior to those of the urea analogues 7. As for the compounds 7, the introduction of chloroethyl as a tail group in the R_2_ position (**7g–i**) led to better efficacy than ethyl or allyl groups (**7a–f**). Cyclizations of 7g–i were also carried out, which only caused the loss rather than an increase of bioactivity (**7j–k**). 

In Table 2, the analogues substituting (thio)urea for guanidine all displayed weak capability in down-regulating leptin level in 3T3-L1 adipocytes, which further demonstrated that the urea moiety was very important for the pharmaceutical activity. Moreover, two bridges, more specifically, 4-aminopiperidine and 4-hydroxypiperidine (**14a–d**, Table 3) were compared and the 4-aminopiperidine linker turned out to be better than 4-hydroxypiperidine for the bioactivity. 

Finally, regarding the role of substituent groups in the phenyl ring R_1_ position, it seemed that the compounds with a trifluoromethyl group always displayed better activities than those with other substituents, which might because of the special effect contributed by this group on compounds’ physicochemical and conformational properties [30].

### 2.3. Activity in Diet-Induced Obesity (DIO) Mice

**Figure 2:**
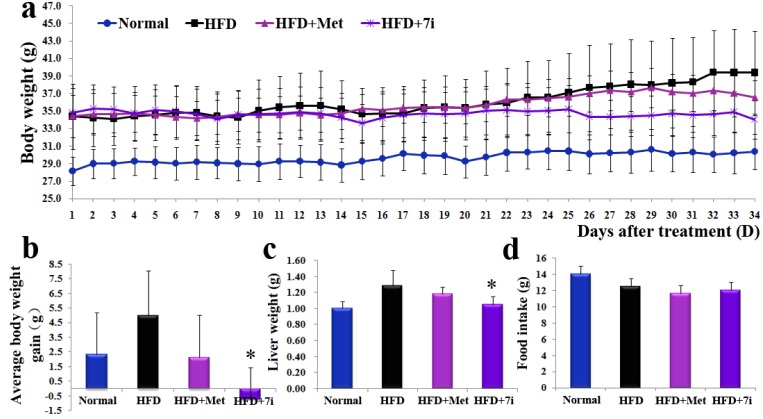
(**a**) Body weights of DIO mice, either HFD group (*n* = 5) or treated groups (*n* = 5), were monitored during the oral administration of metformin (150 mg/kg/day) and 7i (50 mg/kg/day) for 5 weeks; (**b**) Average body weight gain; (**c**) liver weight, and (**d**) Food intake: *, *p* < 0.05, **, *p* < 0.01, *vs.* HFD.

**Figure 3:**
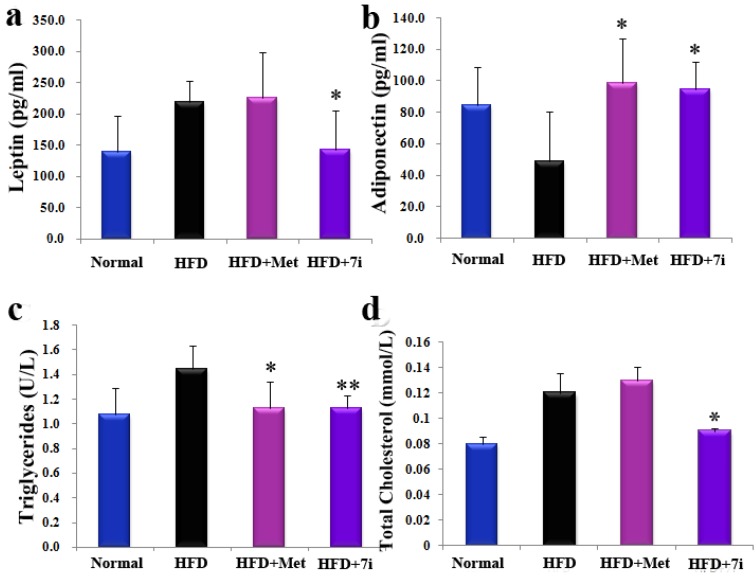
(**a**) Serum leptin level of DIO mice; (**b**) Serum adiponectin level of DIO mice; (**c**) Serum triglyceride; (**d**) total cholesterol: *, *p* < 0.05, **, *p* < 0.01, *vs.* HFD.

Given the excellent *in vitro* leptin level lowering capability of **7i**, it was chosen to evaluate the *in vivo* activity in a diet-induced obesity (DIO) model. DIO is associated with hyperleptinemia and hepatic steatosis in C57Bl/6J mice [31,32]. After oral administration with **7i** once daily at 50 mg/kg for five weeks, the average body weight and liver weight was reduced by 13.5% and 18.4%, respectively, despite the consistent food intake compared to the untreated high-fat diet (HFD) group (Figure 2). Subsequently, corresponding with the screening result in 3T3-L1 adipocytes, **7i** notably reduced the serum level of leptin by 34.6%, as well as TG, total cholesterol (TC), LDL-c and HDL-c which were respectively reduced by 17.3%, 25.0%, 57.7%, and 20.9 %. Adiponectin, which counteracted the effect of leptin in aggravating IR, is considered ia protector in hepatic steatosis and NASH [33,34,35]. The *in vivo* test showed that the serum level of adiponectin was remarkably elevated by 93.0% after treatment with **7i**. Nevertheless the changes of aspartate aminotransferase (AST), alanine transaminase (ALT) and total protein (TP) remained in a normal range, which suggested **7i** scarcely causes any serious hepatotoxicity (Figure 3, Table 4).

**Table 4:** Parameters of Serum Biochemical Markers from DIO mice ^a^_._

Parameter	Normal	HFD	HFD + Met	HFD + 7i
LDL-c (mM)	0.2	0.3	0. 2 ± 0. 1	0.1 **
HDL-c (mM)	1.6 ± 0.4	2.8 ± 0.6	3.0 ± 0.9	2.2 *
ALT (U/L)	35.0 ± 12.7	44.0 ± 9.6	34.0 ± 17.8	36.0 ± 9.6
AST (U/L)	96.0 ± 30.1	105.0 ± 10.0	121.0 ± 30.9	126.0 ± 23.6
TP (g/L)	51.9 ± 3.3	53.1 ± 3.4	57.1 ± 6.3	55.8 ± 3.7

^a^ The HFD-fed (*n* = 5) or treated groups (*n* = 5) were respectively monitored during the oral administration of Met (150 mg/kg/day) and **7i** (50 mg/kg/day) for 5 weeks. The results were expressed as the mean ± SD: *, *p* < 0.05, **, *p* < 0.01, *vs.* HFD.

### 2.4. Histopathological Evaluation

**Figure 4:**
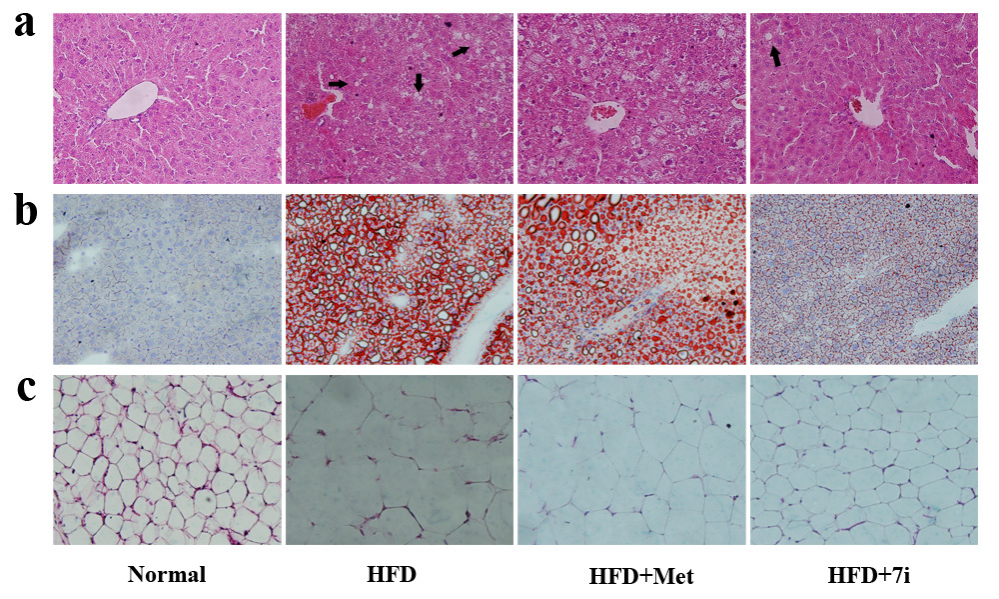
(**a**), (**b**) H&E staining and Oil Red O of liver tissue (scale, 200×). (**c**) H&E stainingof fat tissue from DIO rats (scale, 100×). Lipid droplets are marked using black arrowheads.

The effect of **7i** in protecting the liver from the progression of obesity-related fat accumulation and hepatic steatosis was further verified by hematoxylin-eosin (H&E) and Oil Red O staining assay [36,37]. As shown in Figure 4, DIO in C57/Bl6 mice is accompanied by an obvious fat deposition (steatosis) and a remarkable increase in adipocyte size in the liver tissues compared to the normal group, while a remarkable reduction of lipid droplets (black arrowheads) was observed, which means that there was an obvious restriction of the fat accumulation in the section of liver tissue after treatment with **7i** (Figure 4a,b). Meanwhile, the adipocytes were smaller in size than those of the model group and tended to normal levels (Figure 4c). The metformin-treated group showed weaker potency in ameliorating both the fat deposition and the size of adipocytes compared to **7i**. 

## 3. Experimental

### 3.1. General Information

Chemistry reagents of analytical grade were purchased from Changzheng Chemical Factory, (Chengdu, China). TLC was performed on 0.20 mm Silica Gel 60 F_254_ plates (Qingdao Ocean Chemical Factory, Shandong, China). ^1^H- and ^13^C-NMR spectra were recorded at 400 MHz and 100 MHz, respectively, on a Varian model Gemini 400 spectrometer (Varian, Palo Alto, CA, USA). Chemical shifts (δ) are quoted in ppm relative to tetramethylsilane (TMS) as an internal standard, where (δ) TMS = 0.00 ppm. The multiplicity of the signal is indicated as s, singlet; d, doublet; t, triplet; q, quartet; m, multiplet, defined as all multipeak signals where overlap or complex coupling of signals makes definitive descriptions of peaks difficult. Mass Spectra (MS) were measured on a Q-TOF Premier mass spectrometer (Micromass, Manchester, UK) electrospray ionization (ESI).

### 3.2. Chemistry

#### 3.2.1. Synthesis of *tert*-Butyl (1-(4-nitrobenzyl)piperidin-4-yl)carbamate (**3**)

4-(*tert*-Butoxycarbonylamino)piperidine (10.5 mmol), nitrobenzyl bromide (10.0 mmol), and potassium carbonate (30.0 mmol) in acetone (60 mL) were stirred at 60 °C for 4 h. After the reaction was complete, the mixture was concentrated under reduced pressure, and the residue was poured into water (100 mL) to afford a pure light yellow solid which was collected by filtration, washed with water, and then dried for the further use without any additional purification. Yield 95.2%; Yellow solid; ^1^H-NMR (DMSO-*d_6_*): δ 8.18 (d, 2H, *J* = 8.4 Hz), 7.51 (d, 2H, *J* = 8.0 Hz), 4.44 (m, 1H), 3.58–3.49 (m, 3H), 2.77 (m, 2H), 2.15 (m, 2H), 1.95–1.92 (m, 2H), 1.44–1.42 (m, 11H); MS (ESI), *m/z*: 336.18 [M + H]^+^_._

#### 3.2.2. General Procedure for the Synthesis of Compounds **4**

To a solution of **3** (1.0 mmol) in DCM (1 mL) was added TFA (1 mL) at 0 °C. The resulting brownish orange solution is warmed to room temperature and stirred for 3 h and then concentrated *in vacuo* to afford a brown oil. The residue was treated with diethyl ether (50 mL) to yield a white solid which was isolated by filtration.

#### 3.2.3. General Procedure for the Synthesis of Compounds **5** and **12**

To a solution of substituted benzoic acid (2.0 mmol), EDCI (1.5 mmol) and DMAP (1.0 mmol) in DCM (10 mL), **4** (2.0 mmol) was added and the mixture stirred at room temperature for 14 h. The reaction mixture was then extracted with DCM and water, and the organic layer was washed with brine and dried over anhydrous sodium sulfate. The solvent was removed under reduced pressure. The residue was purified by column chromatography on silica gel to afford the title compounds.

#### 3.2.4. General Procedure for the Synthesis of Compounds **6** and **13**

A solution of the **5** (1 mmol) in anhydrous EtOH was treated with solid SnCl_2_·2H_2_O (7 mmol). The mixture was stirred at 80 °C for 1.5 h and then poured cautiously into saturated aqueous K_2_CO_3_. The resulting gelatinous emulsion was filtered through a pad of Celite, washed with DCM and the biphasic filtrate was extracted with DCM. The combined organic extracts were dried (Na_2_SO_4_) and concentrated *in vacuo*, affording the anilines without further purification.

*N-(1-(4-Aminobenzyl)piperidin-4-yl)-3-(trifluoromethyl)benzamide* (**6a**). Yield 75.2%; Yellow solid; ^1^H-NMR (DMSO-*d_6_*): δ 8.02 (s, 1H), 7.93 (d, 1H, *J* = 7.6 Hz), 7.75 (d, 1H, *J* = 7.6 Hz), 7.57 (t, 1H, *J* = 8.0 Hz), 7.12 (d, 2H, *J* = 8.0 Hz), 6.66 (d, 2H, *J* = 8.0 Hz), 6.17 (d, 2H, *J* = 7.6 Hz), 4.05–4.02 (m, 1H), 3.50 (s, 2H), 2.95 (m, 2H), 2.22 (m, 2H), 2.03 (m, 2H), 1.71–1.65 (m, 2H); MS (ESI), *m/z*: 378.17 [M + H]^+^.

*N-(1-(4-Aminobenzyl)piperidin-4-yl)-3-fluoro-4-(trifluoromethyl)benzamide* (**6b**). Yield 66.8%; Yellow solid; ^1^H-NMR (DMSO-*d_6_*): δ 7.69–7.58 (m, 3H), 7.12 (d, 2H, *J* = 8.0 Hz), 7.66 (d, 2H, *J* = 8.4 Hz), 6.13 (m, 1H), 4.03–4.01 (m, 1H), 3.67 (s, 2H), 2.94 (m, 2H), 2.12 (m, 2H), 2.03 (m, 2H), 1.68 (m, 2H); MS (ESI), *m/z*: 396.16 [M + H]^+^.

*N-(1-(4-Aminobenzyl)piperidin-4-yl)-4-chloro-2-methoxybenzamide* (**6c**). Yield 65.2%; Yellow solid; ^1^H-NMR (DMSO-*d_6_*): δ 8.16 (d, 2H, *J* = 2.8 Hz), 7.77 (d, 1H, *J* = 7.6 Hz), 7.37 (dd, 1H, *J* = 8.8 Hz, *J* = 2.8 Hz), 7.85–7.81 (m, 1H), 7.10 (d, 1H, *J* = 8.0 Hz), 6.90 (d, 1H, *J* = 8.8 Hz), 6.65 (d, 2H, *J* = 8.4 Hz), 4.05–4.03 (m, 1H), 3.91 (s, 3H), 3.64 (brs, 2H), 3.42 (s, 2H), 2.78 (m, 2H), 2.18 (m, 2H), 2.02–1.98 (m, 2H), 1.57–1.55 (m, 2H); MS (ESI), *m/z*: 374.16 [M + H]^+^.

*N-(1-(4-Aminobenzyl)piperidin-4-yl)-2,2-difluorobenzo[d]*[1,3]*dioxole-4-carboxamide* (**6d**)*.* Yield 63.0%; Yellow solid; ^1^H-NMR (DMSO-*d_6_*): δ 7.67 (dd, 1H, *J* = 6.4 Hz, *J* = 2.8 Hz), 7.11–7.07 (m, 4H), 6.59 (d, 2H, *J* = 8.0 Hz), 6.50 (d, 2H, *J* = 7.6 Hz), 4.02 (m, 1H), 3.50 (s, 2H), 2.92 (d, 2H, *J* = 9.6 Hz), 2.25 (t, 2H, *J* = 10.8 Hz), 2.00 (d, 2H, *J* = 10.4 Hz), 1.77–1.72 (m, 2H); MS (ESI), *m/z*: 390.16 [M + H]^+^.

*N-(1-(4-Aminobenzyl)piperidin-4-yl)-3-(1,1,2,2-tetrafluoroethyl)benzamide* (**6e**). Yield 65.2%; Yellow solid; ^1^H-NMR (DMSO-*d_6_*): δ 7.64–7.62 (m, 2H), 7.45 (t, 1H, *J* = 8.0 Hz), 7.13 (d, 2H, *J* = 7.6 Hz), 6.66 (d, 2H, *J* = 7.6 Hz), 6.09–5.80 (m, 2H), 4.02 (m, 1H), 3.68 (s, 2H), 2.97 (d, 2H, *J* = 10.0 Hz), 2.25 (t, 2H, *J* = 12.0 Hz), 2.04 (d, 2H, *J* = 11.2 Hz), 1.74–1.72 (m, 2H); MS (ESI), *m/z*: 410.18 [M + H]^+^.

*N-(1-(4-Aminobenzyl)piperidin-4-yl)-4-(difluoromethoxy)benzamide* (**6f**). Yield 76.2%; Yellow solid; ^1^H-NMR (DMSO-*d_6_*): δ 7.91 (d, 2H, *J* = 8.4 Hz), 7.45 (d, 2H, *J* = 6.4 Hz), 7.20 (d, 1H, *J* = 8.0 Hz), 7.13 (d, 2H, *J* = 8.4 Hz), 6.83–6.47 (m, 3H), 4.06 (brs, 2H), 3.73 (s, 2H), 2.92 (d, 2H, *J* = 10.8 Hz), 2.48 (m, 2H), 2.03–1.99 (m, 24H); MS (ESI), *m/z*: 376.18 [M + H]^+^.

*N-(1-(4-Aminobenzyl)piperidin-4-yl)nicotinamide* (**6g**). Yield 45.2%; Yellow solid; ^1^H-NMR (DMSO-*d_6_*): δ 8.55 (d, 2H, *J* = 4.4 Hz), 8.17 (d, 1H, *J* = 8.0 Hz), 8.03 (d, 1H, *J* = 7.2 Hz), 7.85–7.81 (m, 1H), 7.43–7,20 (m, 1H), 7.20 (d, 2H, *J* = 7.2 Hz), 6.69 (d, 2H, *J* = 8.4 Hz), 5.99 (m, 1H), 4.04 (m, 1H), 3.65 (m, 4H), 3.07 (m, 2H), 2.41–2.39 (m, 2H); MS (ESI), *m/z*: 310.18 [M + H]^+^.

*N-(1-(4-Aminobenzyl)piperidin-4-yl)-5-methylpyrazine-2-carboxamide* (**6h**). Yield 75.2%; Yellow solid; ^1^H-NMR (DMSO-*d_6_*): δ 9.25 (s, 1H), 8.37 (s, 1H), 7.66 (d, 2H, *J* = 8.0 Hz), 7.10 (d, 2H, *J* = 8.4 Hz), 6.65 (d, 2H, *J* = 8.4 Hz), 4.04–3.96 (m, 1H), 3.65 (brs, 2H), 3.43 (s, 2H), 2.86 (d, 2H, *J* = 11.2 Hz), 2.64 (s, 2H), 2.17 (t, 2H, *J* = 11.2 Hz), 1.99 (d, 2H, *J* = 10.8 Hz), 1.67–1.58 (m, 2H); MS (ESI), *m/z*: 326.19 [M + H]^+^.

*(4-(4-Aminophenoxy)piperidin-1-yl)(3-fluoro-4-(trifluoromethyl)phenyl) methanone* (**13a**)*.* Yield 55.2%; Yellow solid; ^1^H-NMR (DMSO-*d_6_*): δ 7.67 (t, 1H, *J* = 7.6 Hz), 7.29–7.24 (m, 2H), 6.77 (d, 2H, *J* = 8.4 Hz), 6.64 (d, 2H, *J* = 8.4 Hz), 4.44–4.39 (m, 1H), 3.85 (s, 2H), 3.61 (m, 1H), 3.31 (m, 1H), 1.94–1.80 (m, 4H); MS (ESI), *m/z*: 383.13 [M + H]^+^.

*4-((1-((4-Fluorophenyl)sulfonyl)piperidin-4-yl)oxy)aniline* (**13b**). Yield 60.2%; Yellow solid; ^1^H-NMR (DMSO-*d_6_*): δ 7.81–7.77 (m, 2H), 7.62–7.53 (m, 1H), 7.25–7.21 (m, 1H), 6.66–6.63 (m, 2H), 6.61–6,57 (m, 4H) 4.22–4.18 (m, 1H), 3.17–3.10 (m, 1H), 1.97–1.87 (m, 4H); MS (ESI), *m/z*: 351.11 [M + H]^+^.

#### 3.2.5. General Procedure for the Synthesis of Compounds **7a–7i**, **8**, **14a**, **14b**, **14d**

Ethyl isocyanate (3.6 mmol) was added dropwise to a stirred solution of the relevant aniline **6** or **13** (3 mmol) in DCM (15 mL). The reaction mixture was stirred overnight at ambient temperature. The resulting crude precipitate was filtered, washed with cold ether, and purified by recrystallization from ethanol and water. 

*N-(1-(4-(3-Ethylureido)benzyl)piperidin-4-yl)-3-(trifluoromethyl)benzamide* (**7a**)*.* Yield 55.2%; White solid; ^1^H-NMR (DMSO-*d_6_*): δ 8.52 (d, 1H, *J* = 8.0 Hz), 8.35 (m, 2H), 8.15 (s, 1H), 7.99 (d, 1H, *J* = 8.0 Hz), 7.71 (t, 2H, *J* = 8.0 Hz), 7.32 (d, 2H, *J* = 8.0 Hz), 7.13 (d, 2H, *J* = 8.0 Hz), 6.05 (t, 1H, *J* = 4.0 Hz), 3.77 (m, 1H), 3.37 (s, 2H), 3.06 (m, 2H), 2.82 (d, 2H, *J* = 12.0 Hz), 1.98 (t, 2H, *J* = 12.0 Hz), 1.77 (m, 2H), 1.55 (m, 2H), 1.04 (t, 2H, *J* = 8.0 Hz); HRMSMS (ESI), *m/z*: 449.2086 [M + H]^+^_._

*N-(1-(4-(3-Allylureido)benzyl)piperidin-4-yl)-3-(trifluoromethyl)benzamide* (**7b**). Yield 54.9%; White solid; ^1^H-NMR (DMSO-*d_6_*): δ 8.52 (d, 1H, *J* = 4.0 Hz), 8.45 (s, 1H), 8.17–8.13 (m, 1H), 7.89 (d, 1H, *J* = 8.0 Hz), 7.71–7.59 (m, 1H), 7.33 (d, 2H, *J* = 8.0 Hz), 7.14 (d, 2H, *J* = 8.0 Hz), 6.22 (m, 1H), 5.87–5.85 (m, 1H), 5.16 (d, 1H, *J* = 16.0 Hz), 5.06 (d, 1H, *J* = 12.0 Hz), 3.77–3.72 (m, 3H), 3.37 (s, 2H), 2.82 (d, 2H, *J* = 12.0 Hz), 1.99 (t, 2H, *J* = 12.0 Hz), 1.78 (m, 2H), 1.58 (t, 2H, *J* = 12.0 Hz); HRMS (ESI), *m/z*: 461.2086 [M + H]^+^_._

*N-(1-(4-(3-Ethylureido)benzyl)piperidin-4-yl)-3-fluoro-4-(trifluoromethyl)benzamide* (**7c**). Yield 44.2%; White solid; ^1^H-NMR (DMSO-*d_6_*): δ 8.55 (d, 1H, *J* = 8.0 Hz), 8.37 (s, 1H), 7.91–7.81 (m, 2H), 7.32 (d, 1H, *J* = 8.0 Hz), 6.07–6.05 (m, 1H), 3.75 (m, 2H), 3.09–3.06 (m, 2H), 2.80 (m, 2H), 1.99 (m, 2H), 1.77 (m, 2H), 1.57–1.54 (m, 2H) 1.04 (t, 2H, *J* = 8.0 Hz); HRMS (ESI), *m/z*: 467.1992 [M + H]^+^_._

*N-(1-(4-(3-Ethylureido)benzyl)piperidin-4-yl)-5-methylpyrazine-2-carboxamide* (**7d**). Yield 47.8%; White solid; ^1^H-NMR (CD_3_OD): δ 9.07 (s, 1H), 8.57 (s, 1H), 7.49 (d, 2H, *J* = 8.4 Hz), 7.39 (d, 2H, *J* = 8.4 Hz), 4.23 (m, 3H), 3.48 (m, 3H), 3.23 (q, 2H, *J* = 7.2 Hz ), 3.13 (m, 2H), 2.63 (s, 3H), 2.20–2.17 (m, 2H), 2.01–1.98 (m, 2H) 1.15 (t, 2H, *J* = 7.2 Hz); HRMS (ESI), *m/z*: 397.2274 [M + H]^+^_._

*4-Chloro-N-(1-(4-(3-ethylureido)benzyl)piperidin-4-yl)-2-methoxybenzamide* (**7e**)*.* Yield 39.5%; White solid; ^1^H-NMR (DMSO-*d_6_*): δ 7.75 (d, 1H, *J* = 2.4 Hz), 7.45 (dd, 1H, *J* = 8.8 Hz, *J* = 2.8 Hz), 7.33 (d, 2H, *J* = 8.4 Hz), 7.22 (d, 2H, *J* = 8.4 Hz), 7.12 (d, 1H, *J* = 8.8 Hz), 3.92 (s, 4H), 3.50 (s, 2H), 3.22 (q, 2H, *J* = 7.2 Hz), 2.84 (d, 2H, *J* = 10.0 Hz), 2.24 (m, 2H), 1.96 (d, 2H, *J* = 10.0 Hz), 1.62 (m, 2H), 1.14 (t, 2H, *J* = 11.2 Hz); HRMS (ESI), *m/z*: 445.1928 [M + H]^+^_._

*N-(1-(4-(3-Allylureido)benzyl)piperidin-4-yl)-4-chloro-2-methoxybenzamide* (**7f**). Yield 57.7%; White solid; ^1^H-NMR (CD_3_OD): δ 7.84 (d, 1H, *J* = 4.0 Hz), 7.44 (dd, 1H, *J* = 8.8 Hz, *J* = 2.8 Hz), 7.35 (d, 2H, *J* = 8.8 Hz), 7.22 (d, 2H, *J* = 8.8 Hz), 7.08 (d, 1H, *J* = 8.8 Hz), 5.93–5.87 (m, 1H), 5.22 (dd, 1H, *J* = 17.2 Hz, *J* = 1.6 Hz), 5.11 (dd, 1H, *J* = 17.2 Hz, *J* = 1.6 Hz), 3.93 (s, 3H), 3.82 (d, 2H, *J* = 5.2 Hz), 3.51 (s, 2H), 2.84 (d, 2H, *J* = 9.6 Hz), 2.24 (t, 2H, *J* = 9.6 Hz), 1.98 (d, 2H, *J* = 10.8 Hz), 1.62 (q, 2H, *J* = 10.0 Hz); HRMS (ESI), *m/z*: 457.1928 [M + H]^+^_._

*4-Chloro-N-(1-(4-(3-(2-chloroethyl)ureido)benzyl)piperidin-4-yl)-2-methoxybenzamide* (**7g**). Yield 60.2%; White solid; ^1^H-NMR (DMSO-*d_6_*): δ 8.62 (s, 1H), 8.06 (d, 1H, *J* = 7.6 Hz), 7.59 (d, 1H, *J* = 2.8 Hz), 7.49 (dd, 1H, *J* = 8.4 Hz, *J* = 2.8 Hz), 7.34 (d, 2H, *J* = 8.4 Hz), 7.16–7.14 (m, 3H), 6.39 (t, 1H, *J* = 5.6 Hz), 3.86 (s, 3H), 3.83–3.77 (m, 1H), 3.65 (t, 2H, *J* = 6.4 Hz), 2.72 (d, 2H, *J* = 10.4 Hz), 2.06 (m, 2H), 1.77 (m, 2H), 1.51 (m, 2H); HRMS (ESI), *m/z*: 479.1538 [M + H]^+^_._

*N-(1-(4-(3-(2-Chloroethyl)ureido)benzyl)piperidin-4-yl)-3-fluoro-4-(trifluoromethyl)benzamide* (**7h**). Yield 44.5%; White solid; ^1^H-NMR (DMSO-*d_6_*): δ 9.05 (s, 1H), 8.85 (bs, 1H), 7.95–7.90 (m, 2H), 7.48–7.42 (m, 2H), 6.61 (m, 1H), 4.11–3.99 (m, 2H), 3.66 (m, 2H), 3.42 (m, 2H), 3.02 (m, 2H), 1.94 (m, 3H), 1.20 (m, 2H); HRMS (ESI), *m/z*: 501.1602 [M + H]^+^_._

*N-(1-(4-(3-(2-Chloroethyl)ureido)benzyl)piperidin-4-yl)-3-(trifluoromethyl)benzamide* (**7i**). Yield 50.4%; White solid; ^1^H-NMR (DMSO-*d_6_*): δ 8.66 (s, 1H), 8.54 (d, 1H, *J* = 8.0 Hz), 8.18–8.14 (m, 2H), 7.90 (d, 1H, *J* = 4.0 Hz), 7.69 (t, 1H, *J* = 8.0 Hz), 7.35 (d, 2H, *J* = 8.0 Hz), 7.17 (d, 2H, *J* = 8.0 Hz), 6.41 (s, 1H), 3.79 (s, 2H), 3.66 (m, 2H), 3.42 (m, 3H), 2.85 (s, 2H), 2.01 (s, 2H), 1.80 (m, 2H), 1.61 (m, 2H); HRMS (ESI), *m/z*: 483.1772 [M + H]^+^_._

*N-(1-(4-(3-Ethylthioureido)benzyl)piperidin-4-yl)-3-(trifluoromethyl)benzamide* (**8a**). Yield 64.2%; White solid; ^1^H-NMR (CDCl_3_): δ 8.01 (s, 1H), 7.94 (d, 1H, *J* = 8.0 Hz), 7.93 (m, 2H), 7.55 (m, 2H), 7.39 (d, 1H, *J* = 8.0 Hz), 7.19 (d, 2H, *J* = 8.0 Hz), 6.12–6.07 (m, 2H), 4.04 (m, 1H), 3.69–3.66 (m, 2H), 3.56 (s, 2H), 2.92 (d, 1H, *J* = 12.0 Hz), 2.28–2.23 (m, 3H), 2.06 (d, 2H, *J* = 12.0 Hz), 1.67–1.64 (m, 2H), 1.20 (t, 2H, *J* = 8.0 Hz); HRMS (ESI), *m/z*: 465.1858 [M + H]^+^_._

*N-(1-(4-(3-Ethylthioureido)benzyl)piperidin-4-yl)-3-fluoro-4-(trifluoromethyl)benzamide* (**8b**). Yield 35.2%; White solid; ^1^H-NMR (CDCl_3_): δ 7.62–7.60 (m, 3H), 7.29 (d, 1H, *J* = 8.0 Hz), 7.17 (d, 1H, *J* = 8.0 Hz), 3.93–3.92 (m, 1H), 3.58 (q, 2H, *J* = 8.0 Hz), 3.50 (s, 2H), 2.90 (d, 1H, *J* = 12.0 Hz), 2.15 (m, 2H), 1.92 (d, 2H, *J* = 12.0 Hz), 1.65–1.55 (m, 2H), 1.13 (t, 2H, *J* = 8.0 Hz); HRMS (ESI), *m/z*: 483.1763 [M + H]^+^_._

*4-Chloro-N-(1-(4-(3-ethylthioureido)benzyl)piperidin-4-yl)-2-methoxy benzamide* (**8c**). Yield 65.2%; White solid; ^1^H-NMR (DMSO-*d_6_*): δ 9.46 (bs, 1H), 8.12 (d, 1H, *J* = 7.2 Hz), 7.75 (s, 1H), 7.65 (s, 1H), 7.54 (d, 1H, *J* = 7.2 Hz), 7.38 (d, 2H, *J* = 8.0 Hz), 7.28 (d, 2H, *J* = 8.0 Hz), 7.20 (d, 1H, *J* = 8.0 Hz), 3.91–3.82 (m, 4H), 3.47–3.42 (m, 4H), 2.79 (d, 2H, *J* = 9.6 Hz), 2.13 (m, 2H), 1.87 (m, 2H), 1.57 (m, 2H), 1.16 (t, 2H, *J* = 6.8 Hz); HRMS (ESI), *m/z*: 461.1700 [M + H]^ +^_._

*1-Ethyl-3-(4-((1-(3-fluoro-4-(trifluoromethyl)benzoyl)piperidin-4-yl)oxy)phenyl)urea* (**14a**). Yield 35.2%; White solid; ^1^H-NMR (CDCl_3_): δ 7.67 (t, 1H, *J* = 6.8 Hz), 7.19 (d, 2H, *J* = 8.8 Hz), 6.87 (d, 2H, *J* = 8.8 Hz), 6.26 (s, 1H), 4.65–4.56 (m, 2H), 3.87 (m, 2H), 3.47 (m, 2H), 2.87–2.37 (m, 2H), 1.95–1.84 (m, 4H), 1.13 (t, 3H, *J* = 7.2 Hz); HRMS (ESI), *m/z*: 454.1676 [M + H]^+^_._

*1-(2-Chloroethyl)-3-(4-((1-(3-fluoro-4-(trifluoromethyl)benzoyl)piperidin-4-yl)oxy)phenyl)urea* (**14b**). Yield 35.2%; White solid; ^1^H-NMR (CDCl_3_): δ 7.68 (t, 1H, *J* = 6.8 Hz), 7.18 (d, 2H, *J* = 8.0 Hz), 6.86 (d, 2H, *J* = 8.0 Hz), 6.59 (s, 1H), 5.27 (m, 1H), 4.56–4.55 (m, 1H), 3.87 (m, 2H), 3.65–3.63 (m, 2H), 3.59–3.55 (m, 2H), 3.47 (m, 1H), 2.05–1.84 (m, 4H); MS (ESI), *m/z*: 488.13 [M + H]^+^_._

*1-Ethyl-3-(4-((1-((4-fluorophenyl)sulfonyl)piperidin-4-yl)oxy)phenyl)urea* (**14d**). Yield 55.2%; White solid; ^1^H-NMR (DMSO-*d_6_*): δ 8.16 (s, 1H), 7.86–7.83 (m, 1H), 7.76–7.73 (m, 1H), 7.69–7.65 (m, 1H), 7.51 (t, 1H, *J* = 7.2 Hz), 7.21 (dd, 2H, *J* = 8.8 Hz, *J* = 2.8 Hz), 6.74 (dd, 2H, *J* = 8.8 Hz, *J* = 3.2 Hz), 5.94 (s, 1H), 4.31–2.89 (m, 1H), 3.22 (m, 2H), 3.10–3.03 (m, 2H), 2.84 (t, 2H, *J* = 8.8 Hz), 1.93 (m, 2H), 1.65–1.63 (m, 2H), 1.02 (t, 3H, *J* = 7.2 Hz); HRMS (ESI), *m/z*: 422.1472 [M + H]^+^_._

#### 3.2.6. General Procedure for the Synthesis of Compounds **7j**, **7k**, **7l** and **14c**

Sodium hydride (3 mmol) was added slowly to a cold solution of compound **7g**, **7h**, **7i** or **14b** (1 mmol) in THF (1.5 mL) under a nitrogen atmosphere. The ice bath was then removed after 30 min, and the mixture was stirred at room temperature for 5 h. After the reaction was completed, the mixture was diluted with cold water, and the resulting precipitate was filtered, washed with cold water and cold ether, then dried to afford the target compounds without further purification. 

*4-Chloro-2-methoxy-N-(1-(4-(2-oxoimidazolidin-1-yl)benzyl)piperidin-4-yl)benzamide* (**7j**). Yield 55.2%; White solid; ^1^H-NMR (CDCl_3_): δ 8.15 (d, 1H, *J* = 2.4 Hz), 7.77 (d, 1H, *J* = 7.2 Hz), 7.48 (d, 1H, *J* = 8.4 Hz), 7.37 (dd, 1H, *J* = 8.8 Hz, *J* = 2.8 Hz), 7.30 (d, 2H, *J* = 8.4 Hz), 6.90 (d, 2H, *J* = 8.8 Hz), 4.03 (m, 1H), 3.96–3.92 (m, 4H), 3.58 (t, 2H, *J* = 8.0 Hz), 3.50 (s, 2H), 2.79 (m, 2H), 2.22 (t, 2H, *J* = 10.4 Hz), 2.01 (d, 2H, *J* = 10.4 Hz), 1.58 (q, 2H, *J* = 10.4 Hz); HRMS (ESI), *m/z*: 443.1772 [M + H]^+^_._

*3-Fluoro-N-(1-(4-(2-oxoimidazolidin-1-yl)benzyl)piperidin-4-yl)-4-(trifluoromethyl)benzamide* (**7k**). Yield 66.4%; White solid;^ 1^H-NMR (CDCl_3_): δ 8.52 (d, 1H, *J* = 2.4 Hz), 8.17 (s, 1H), 8.14 (d, 1H, *J* = 8.0 Hz), 7.89 (d, 1H, *J* = 7.6 Hz), 7.71 (t, 1H, *J* = 7.6 Hz), 7.10 (d, 2H, *J* = 8.4 Hz), 6.98 (d, 2H, *J* = 8.4 Hz), 3.80–3.76 (m, 1H), 3.36 (s, 2H),3.58 (t, 2H, *J* = 8.0 Hz), 2.79 (m, 2H), 2.84 (d, 2H, *J* = 11.6 Hz), 1.99 (t, 2H, *J* = 10.8 Hz), 1.87–1.77 (m, 2H), 1.61–1.53 (m, 2H); HRMS (ESI), *m/z*: 465.1835 [M + H]^+^_._

*N-(1-(4-(2-Oxoimidazolidin-1-yl)benzyl)piperidin-4-yl)-3-(trifluoromethyl)benzamide* (**7l**). Yield 47.9%; White solid; ^1^H-NMR (DMSO-*d_6_*): δ 8.01 (s, 1H), 7.93 (d, 1H, *J* = 7.6 Hz), 7.75 (d, 1H, *J* = 7.6 Hz), 7.57 (t, 1H, *J* = 7.6 Hz), 7.50 (d, 2H, *J* = 8.4 Hz), 7.30 (d, 2H, *J* = 8.4 Hz), 6.10 (d, 1H, *J* = 7.2 Hz), 4.04–4.02 (m, 1H), 3.95 (t, 2H, *J* = 8.0 Hz), 3.58 (t, 2H, *J* = 8.0 Hz), 2.90 (m, 2H), 2.20 (m, 2H), 2.02 (m, 2H), 1.61 (m, 2H); HRMS (ESI), *m/z*: 447.1930 [M + H]^+^_._

*1-(4-((1-(3-Fluoro-4-(trifluoromethyl)benzoyl)piperidin-4-yl)oxy)phenyl)imidazolidin-2-one* (**14c**). Yield 35.2%; White solid; ^1^H-NMR (CDCl_3_): δ 7.67 (t, 1H, *J* = 7.6 Hz), 7.54 (d, 2H, *J* = 9.2 Hz), 6.91 (d, 2H, *J* = 9.2 Hz), 4.69 (m, 1H), 4.55 (m, 2H), 3.93–3.87 (m, 4H), 3.57 (t, 2H, *J* = 8.0 Hz), 1.97–1.84 (m, 4H); HRMS (ESI), *m/z*: 452.1519 [M + H]^+^_._

#### 3.2.7. General Procedure for the Synthesis of **9**

A solution of *tert*-butyl 4-hydroxypiperidine-1-carboxylate (5.0 mmol) in dry pyridine (15 mL) was stirred at 0 °C for 10 min, and a solution of TsCl (7.5 mmol) in dry pyridine (20 mL) was added slowly. The mixture was stirred at room temperature for 6 h. After the reaction was completed, the mixture was poured into ice water. And then the precipitate was collected by filtration, washed with water, and dried *in vacuo* to afford **9** as a white solid.

#### 3.2.8. General Procedure for the Synthesis of 4-(4-Nitrophenoxy)piperidine (**11**)

A mixture of **9** (5.0 mmol), 4-nitrophenol 10 (5.5 mmol), and K_2_CO_3_ (10.0 mmol) in DMF (15 mL) was heated to 100 °C for 10 h. After the reaction was completed, the K_2_CO_3_ was removed by filtration. The filtrate was poured into ice water and extracted with DCM, saturated NaHCO_3_, and brine, and the combined organic layer was dried with MgSO_4_. Finally, the solvent was removed under reduced pressure to give the crude. A solution of the above crude solid (1.0 mmol) in 1 mL TFA/DCM (v/v = 1/1) was stirred for 3 h at 0 °C, then allowed to warm to room temperature. After the reaction was completed, the solvent was removed under reduced pressure. The residue was slowly made alkaline with 2 N NaOH, then the precipitate formed was collected by filtration and washed with water to afford the crude product **11** without further purification. Yield 89.5%; White solid; ^1^H-NMR (DMSO-*d_6_*): δ 8.20 (d, 2H, *J* = 9.2 Hz), 6.96 (d, 2H, *J* = 8.0 Hz), 4.08 (m, 1H), 3.73–3.67 (m, 2H), 3.41–3.35 (m, 2H), 1.99–1.94 (m, 2H), 1.80–1.77 (m, 2H); MS (ESI), *m/z*: 323.15 [M + H]^+^_._

#### 3.2.9. General Procedure for the Synthesis of **16**

The corresponding amine (2.0 mmol) in DCM was treated with mercury (II) chloride (2.2 mmol), *N,N'*-di(*tert*-butoxycarbonyl)thiourea (2.0 mmol) and TEA (6.3 mmol) at 0 °C. The ice bath was removed after 1h, and the resulting mixture was at room temperature overnight. Then, the reaction mixture was filtered through a pad of Celite to get rid of the mercury sulfide formed. The filter cake was rinsed with DCM. The organic phase was concentrated under vacuum to give a residue that was purified by silica gel column chromatography to afford the title compound.

#### 3.2.10. General Procedure for the Synthesis of **17**

Each of the corresponding Boc-protected precursors (0.5 mmol) was treated with TFA/ DCM (15 mL, v/v = 1/1) acid in DCM for 3 h. After the reaction was completed, the solvent was removed under reduced pressure. The residue was slowly basified with saturated NaHCO_3_, extracted with EtOAc. The organic phase was washed with water and brine, then dried over anhydrous Na_2_SO_4_ and concentrated under vacuum to give a residue that was purified by silica gel column chromatography to afford the title compound.

*N-(1-(4-Guanidinobenzyl)piperidin-4-yl)-3-(trifluoromethyl)benzamide* (**17a**)*.* Yield 30.7%; Yellow solid; ^1^H-NMR (DMSO-d_6_): δ 8.64 (d, 1H, *J* = 7.2 Hz), 8.18 (d, 2H, *J* = 8.0 Hz), 7.90 (d, 2H, *J* = 8.0 Hz), 7.71 (t, 2H, *J* = 8.0 Hz), 7.59 (bs, 3H), 7.36 (d, 2H, *J* = 7.6 Hz), 7.18 (d, 2H, *J* = 7.6 Hz), 3.48 (s, 2H), 2.85 (d, 2H, *J* = 10.8 Hz), 2.09–1.99 (m, 2H), 1.79 (m, 2H), 1.66–1.57 (m, 2H); HRMS (ESI), *m/z*: 420.1933 [M + H]^+^_._

*3-Fluoro-N-(1-(4-guanidinobenzyl)piperidin-4-yl)-4-(trifluoromethyl)benzamide* (**17b**). Yield 45.2%; White solid; ^1^H-NMR (DMSO-d_6_): δ 8.55 (d, 1H, *J* = 7.6 Hz), 7.93–7.85 (m, 3H), 7.11 (d, 2H, *J* = 8.0 Hz), 6.77 (d, 2H, *J* = 8.0 Hz), 3.77 (m, 1H), 3.37 (s, 2H), 2.84 (d, 2H, *J* = 11.6 Hz), 1.99 (t, 2H, *J* = 11.2 Hz), 1.80–1.78 (m, 2H), 1.60–1.54 (m, 2H); HRMS (ESI), *m/z*: 438.1839 [M + H]^+^_._

*4-Chloro-N-(1-(4-guanidinobenzyl)piperidin-4-yl)-2-methoxybenzamide* (**17c**). Yield 35.2%; White solid; ^1^H-NMR (DMSO-d_6_): δ 8.08 (d, 1H, *J* = 7.2 Hz), 7.59 (d, 1H, *J* = 2.4 Hz), 7.49 (dd, 1H, *J* = 8.8 Hz, *J* = 2.4 Hz), 7.23 (t, 2H, *J* = 7.6 Hz), 7.15 (d, 1H, *J* = 8.8 Hz), 6.98 (d, 2H, *J* = 7.6 Hz), 3.86 (s, 3H), 3.77 (m, 1H), 3.41 (s, 2H), 2.75 (d, 2H, *J* = 10.0 Hz), 2.06 (t, 2H, *J* = 10.0 Hz), 1.80–1.77 (m, 2H), 1.04 (q, 2H, *J* = 10.0 Hz); HRMS (ESI), *m/z*: 416.1775 [M + H]^+^_._

*2,2-Difluoro-N-(1-(4-guanidinobenzyl)piperidin-4-yl)benzo[d]*[1,3]*dioxole-4-carboxamide* (**17d**). Yield 25.9%; Yellow solid; ^1^H-NMR (CDCl_3_): δ 7.75–7.72 (m, 1H), 7.41 (d, 2H, *J* = 7.6 Hz), 7.20–7.15 (m, 4H), 6.55 (d, 2H, *J* = 7.6 Hz), 4.04 (m, 1H), 3.56 (s, 2H), 2.86 (d, 2H, *J* = 10.4 Hz), 2.30–2.25 (m, 2H), 1.66 (q, 2H, *J* = 10.0 Hz), 1.60–1.54 (m, 2H); HRMS (ESI), *m/z*: 432.1769 [M + H]^+^_._

*N-(1-(4-Guanidinobenzyl)piperidin-4-yl)-3-(1,1,2,2-tetrafluoroethoxy)benzamide* (**17e**). Yield 25.0%; Yellow solid; ^1^H-NMR (DMSO-d_6_): δ 7.79 (d, 1H, *J* = 6.8 Hz), 7.70 (s, 1H), 7.53–7.43 (m, 4H), 7.26 (d, 2H, *J* = 6.4 Hz), 6.48–6.22 (m, 1H), 3.89 (m, 1H), 3.63 (s, 2H), 3.00 (d, 2H, *J* = 9.2 Hz), 2.22 (t, 2H, *J* = 10.0 Hz), 1.96 (d, 2H, *J* = 10.8 Hz), 1.73–1.70 (m, 2H); MS (ESI), *m/z*: 468.1944 [M + H]^+^_._

*4-(Difluoromethoxy)-N-(1-(4-guanidinobenzyl)piperidin-4-yl)benzamide* (**17f**). Yield 22.4%; White solid; ^1^H-NMR (CD_3_OD): δ 7.76 (d, 2H, *J* = 8.4 Hz), 7.37 (d, 2H, *J* = 7.6 Hz), 7.16 (d, 2H, *J* = 8.4 Hz), 7.10 (d, 2H, *J* = 8.4 Hz), 7.02–6.65 (m, 1H), 3.84–3.77 (m, 1H), 3.53 (s, 2H), 2.90 (d, 2H, *J* = 11.6 Hz), 2.15 (t, 2H, *J* = 11.6 Hz), 1.85 (d, 2H, *J* = 9.6 Hz), 1.64–1.56 (m, 2H); HRMS (ESI), *m/z*: 418.1976 [M + H]^+^_._

*N-(1-(4-Guanidinobenzyl)piperidin-4-yl)nicotinamide* (**17g**). Yield 20.7%; White solid; ^1^H-NMR (CDCl_3_): δ 8.54 (d, 1H, *J* = 4.4 Hz), 8.15 (d, 1H, *J* = 7.6 Hz), 8.01 (d, 1H, *J* = 8.4 Hz), 7.84 (td, 1H, *J* = 7.6 Hz, *J* = 1.6 Hz), 7.44–7.41 (m, 1H), 7.38 (d, 2H, *J* = 8.4 Hz), 7.15 (d, 2H, *J* = 8.0 Hz), 3.98–3.96 (m, 1H), 3.50 (s, 2H), 2.82 (d, 2H, *J* = 11.6 Hz), 2.19 (t, 2H, *J* = 10.8 Hz), 1.99 (d, 2H, *J* = 10.4 Hz), 1.68–1.59 (m, 2H); HRMS (ESI), *m/z*: 353.2012 [M + H]^+^_._

*N-(1-(4-((4,5-Dihydro-1H-imidazol-2-yl)amino)benzyl)piperidin-4-yl)-3-(trifluoromethyl)benzamide* (**17h**). Yield 33.2%; White solid; ^1^H-NMR (CDCl_3_): δ 8.01 (s, 1H), 7.92 (d, 1H, *J* = 7.6 Hz), 7.75 (d, 1H, *J* = 7.6 Hz), 7.56 (t, 1H, *J* = 7.6 Hz), 7.20 (d, 2H, *J* = 8.0 Hz), 6.96 (d, 2H, *J* = 8.0 Hz), 6.08 (d, 1H, *J* = 7.6 Hz), 4.02–4.00 (m, 1H), 3.54 (m, 4H), 3.46 (s, 2H), 2.87 (d, 2H, *J* = 11.6 Hz), 2.16 (t, 2H, *J* = 11.2 Hz), 2.02 (d, 2H, *J* = 11.2 Hz), 1.63–1.54 (m, 2H); HRMS (ESI), *m/z*: 674.1947 [M + H]^+^_._

*N-(1-(4-((4,5-Dihydro-1H-imidazol-2-yl)amino)benzyl)piperidin-4-yl)-3-fluoro-4-(trifluoromethyl)-benzamide* (**17i**). Yield 38.1%; White solid; ^1^H-NMR (CDCl_3_): δ 7.67–7.59 (m, 3H), 7.20 (d, 2H, *J* = 8.0 Hz), 6.95 (d, 1H, *J* = 8.0 Hz), 6.19 (d, 1H, *J* = 7.6 Hz), 3.99 (m, 1H), 3.45 (s, 2H), 2.18–2.12 (m, 2H), 2.01–1.99 (m, 2H), 1.62–1.54 (m, 2H); HRMS (ESI), *m/z*: 692.1853 [M + H]^+^_._

### 3.3. Biological Assay Methods

3.3.1. 3T3-L1 Adipocyte Differentiation 

Marine 3T3-L1 preadipocytes (American Type Culture Collection, Rockville, MD, USA) were plated and grown to two days post confluence in 96 well culture plates in DMEM containing 10% fetal bovine serum; Preadipocytes were induced to differentiate by replacing the medium with serum-containing DMEM containing 0.5 mM methyl-3-isobutylxantine (IBMX), 0.25 mM dexamethasone (DEX) and 1 mg/mL insulin. Two days later, the medium was again changed to serum-containing DMEM that contained insulin but no IBMX or DEX. Two days later, the medium was again changed to the original DMEM containing 10% fetal bovine serum in the absence of any differentiating reagents and was replaced every two days. Full differentiation is usually achieved by 8–12 days.

#### 3.3.2. Expression Levels of Leptin in 3T3-L1 Adipocytes

The twelfth day after differentiation, the culture medium was replaced by DMEM supplemented with chemical series of 3 and 4 (10 mM) and rosiglitazone (Sigma-Aldrich, St. Louis, MO, USA) (10 mM). After 24 h, the expression levels of leptin were measured by commercial kits (Linco Research, St. Charles, MO, USA).

#### 3.3.3. Animal Model and Treatment of Diet-Induced Obesity.

C57BL/6J mice were obtained from Western China Experimental Animal Center and housed individually in a room maintained at 25 °C on a light/dark schedule. For DIO mice, 3-week-old male C57BL/6J mice were fed a high fat diet (HFD, Research Diets, Nanjing, china) *ad libitum* for 8 weeks. Then these animals were randomly assigned to four groups consisting of five mice each. The mice received a normal diet with 18.94% of energy derived from fat, 31.67% from protein, and 49.39% from carbohydrates and received a high fat diet with 60.0% of calories from fat, 20.0% from protein, and 20.0% from carbohydrates. HFD + Met (150 mg/kg/day) and HFD + 7i (50 mg/kg/day) in PEG400 or vehicle were orally administered per day for five weeks. Body weight and food intake were measured per day. And serum levels of biomarkers were analyzed after all animals were sacrificed. The percentages were calculated using the formula [(V_HFD_ − V_treatment_)/V_HFD_] × 100. 

#### 3.3.4. Assay for Serum Biochemical Markers

Serum ALT and AST levels were measured by an automated enzymeassay using commercial kits and Roche automated analyzers (RocheDiagnostics GmbH, Manheim, Germany). Serum levels of TG, LDL cholesterol, HDL-cholesterol, and plasma insulin were determined by radioimmunoassay using commercially available kits (Linco Research).

#### 3.3.5. Histopathological Examination

Liver samples were fixed in 4% buffered formalin and embedded in Tissue-Tek OCT compound (Sakura Finetek USA, Torrance, CA, USA) and paraffin for histological analysis. Formalin-fixed and paraffin embedded section (5 mm) was processed routinely for H&E staining. The OCT-embedded samples were serially sectioned at 4 mm. For the evaluation of fat deposition, the frozen liver sections was stained with Oil red O.

## 4. Conclusions

In this study, twenty eight novel (thio)urea- and guanidine-based derivatives have been synthesized by an easy and practicable synthetic method. Subsequently, a credible screening method was adopted to evaluate their bioactivity and **7i** was proved to be a potential agent against diet-induced hepatic steatosis. The pharmacological evaluation was started with the leptin expression on 3T3-L1 adipocytes, and among the tested compounds, **7i** displayed potent capacity in decreasing leptin levels at 10 μM. The weight of body and liver of high-fat diet induced mice were effectively reduced after oral administration of 7i at a dose of 50 mg/ kg/day for five weeks. At the same time, **7i** modulated serum parameters of leptin, adiponectin, TG, TC, LDL-c and HDL-c to appropriate ranges. Consistent with the reduction of liver weight, further H&E and Oil Red O staining confirmed **7i** reduced fat deposition in the liver tissue and restricted the size of adipocytes against fatty liver disease. In summary, these compelling results suggested that **7i** improved the progression of obesity-related hepatic dysfunctions and protected the liver tissue in diet-induced obese mice. Research on the detailed pharmacokinetics and action of mechanism of **7i** are in progress. 

## Supplementary Materials

Supplementary materials can be accessed at: http://www.mdpi.com/1420-3049/19/5/6163/s1.
