# Supplementary material for: Synthesis and Biological Evaluation of Novel Urea- and Guanidine-Based Derivatives for the Treatment of Obesity-Related Hepatic Steatosis
Source: Molecules. 2014 May 15;19(5):6163–83. doi: 10.3390/molecules19056163 (PMC6271706; doi:10.3390/molecules19056163)
Supplement: Supplementary file 1 [file molecules-19-06163-s001.pdf]

Supplementary Materials

Table of Contents

1. The ^{1}H-NMR spectra of compounds **7**, **8**, **14**, **17**
2. The ^{13}C-NMR and HPLC spectra of **7i**

1. The ^{1}H-NMR spectrum of compounds **7**, **8**, **14**, **17**

Figure S1. ^{1}H-NMR of compound **7a** in DMSO-d_6.

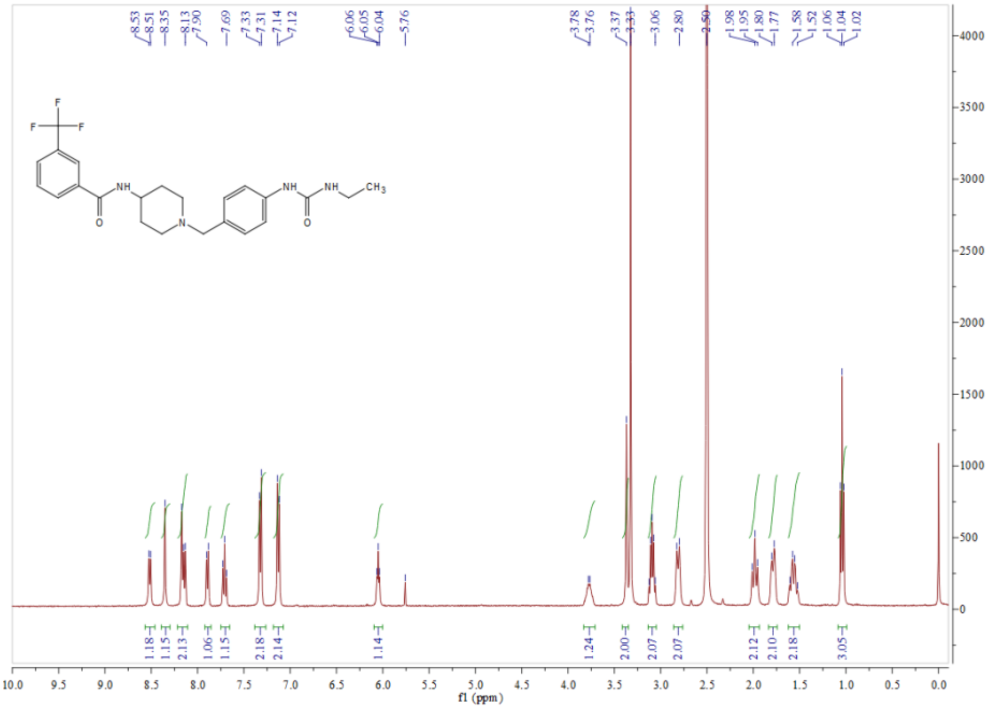

Figure S2. ^{1}H-NMR of compound **7b** in DMSO-d_6.

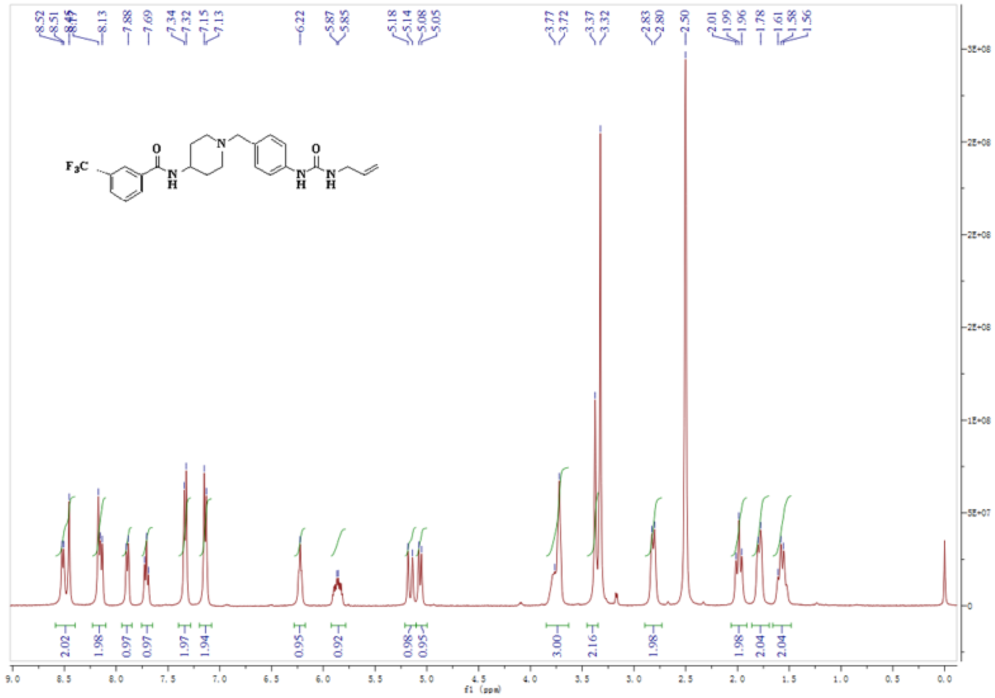

Figure S3. ^{1}H-NMR of compound **7c** in DMSO-d_6.

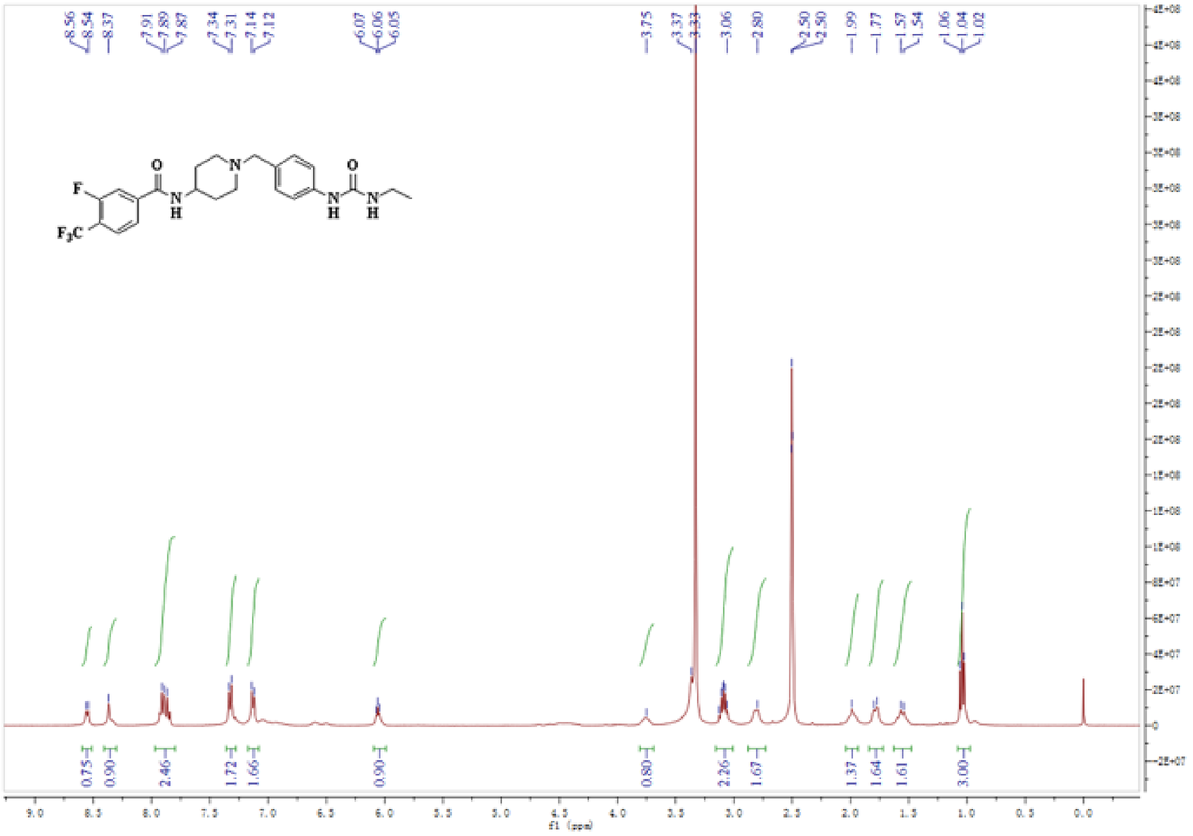

Figure S4. ^{1}H-NMR of compound **7d** in CD_3OD.

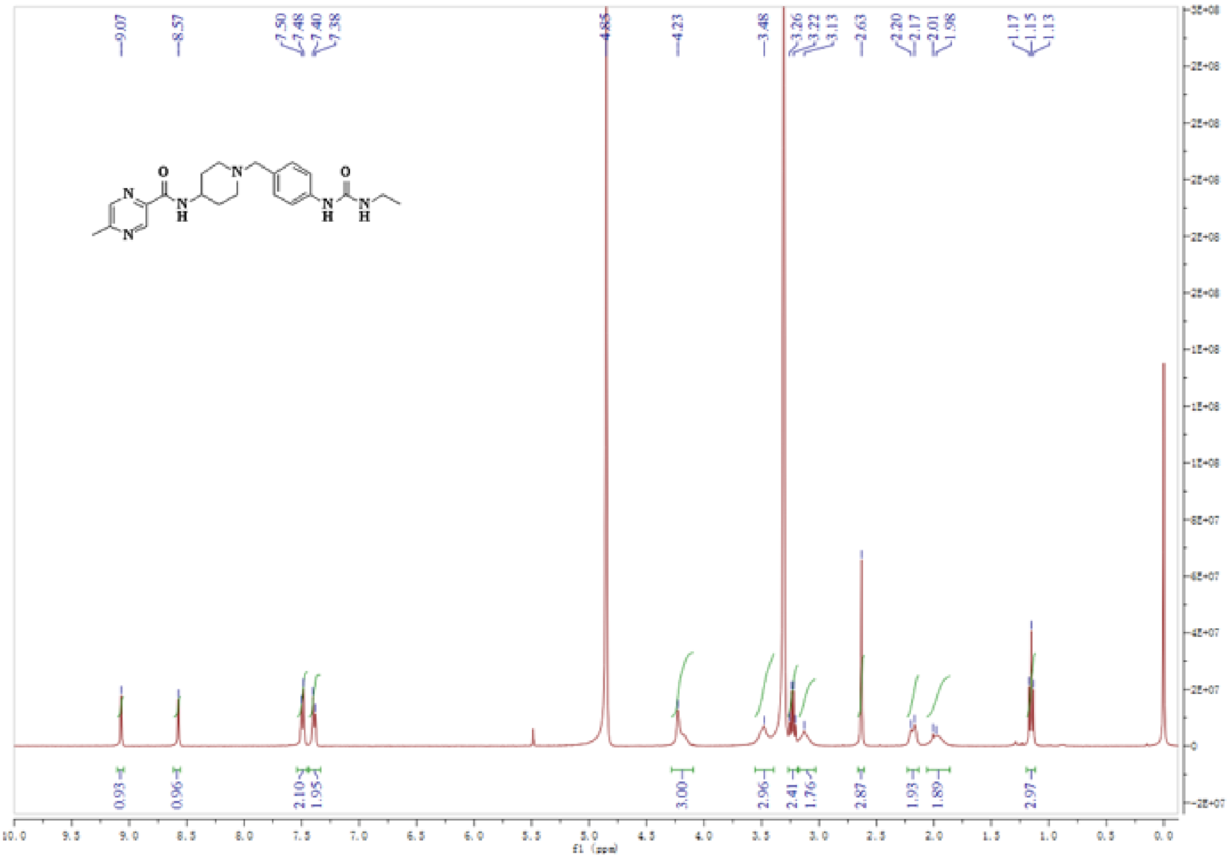

Figure S5. ^{1}H-NMR of compound **7f** in CD_3OD.

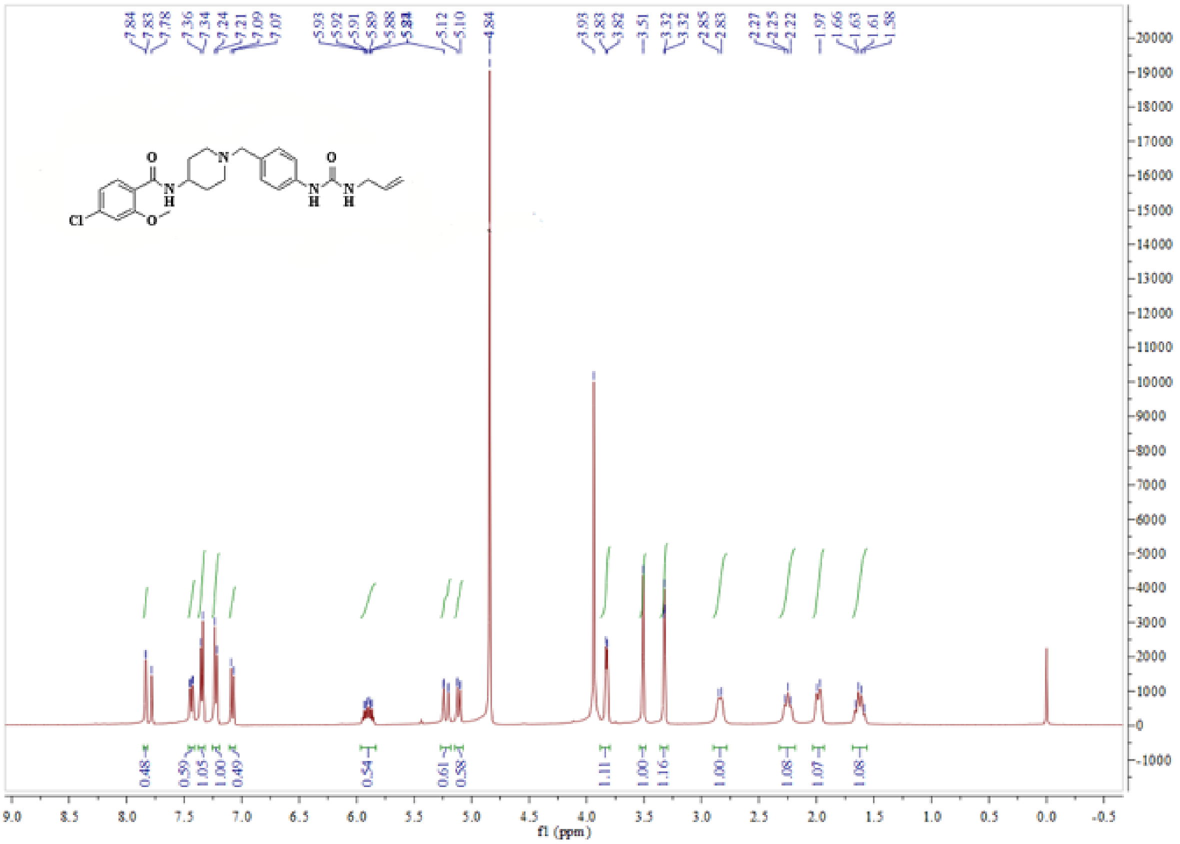

Figure S6. ^{1}H-NMR of compound **7g** in DMSO-d_6.

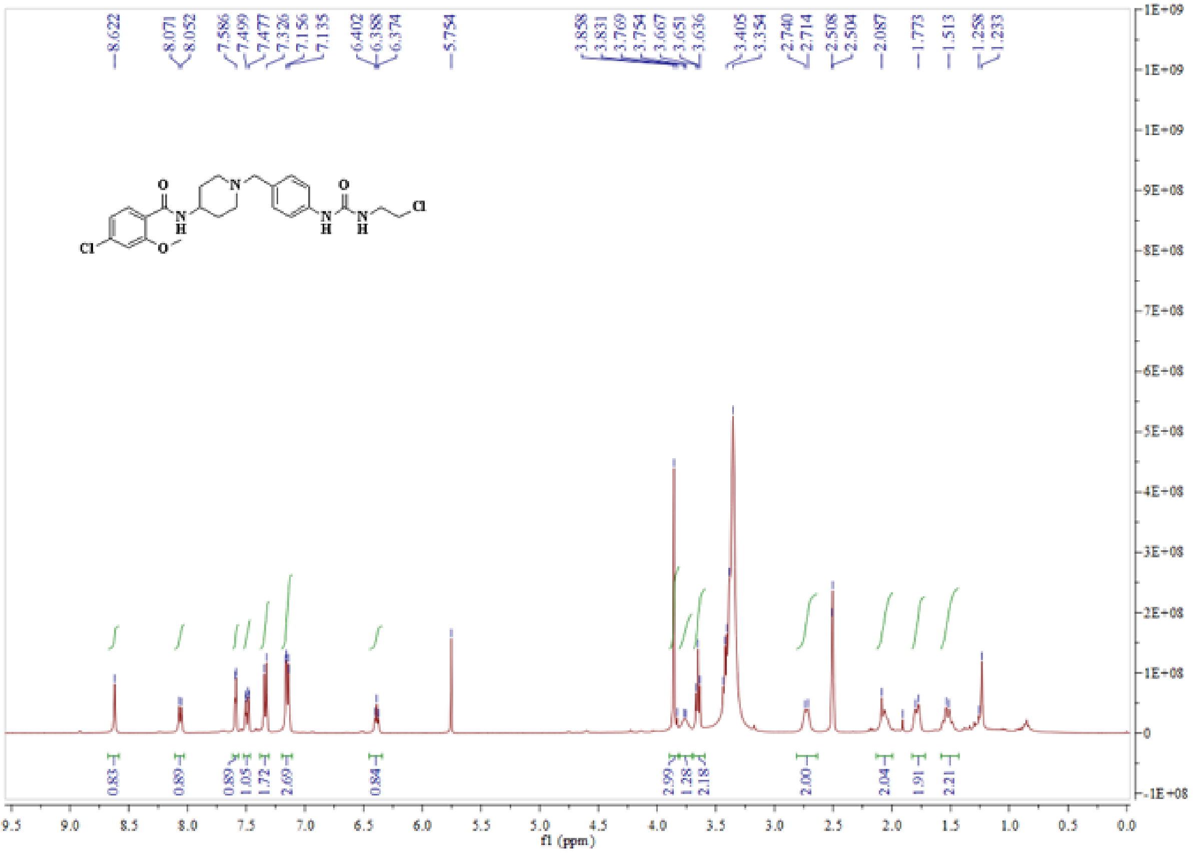

Figure S7. ^{1}H-NMR of compound **7h** in DMSO-d_6.

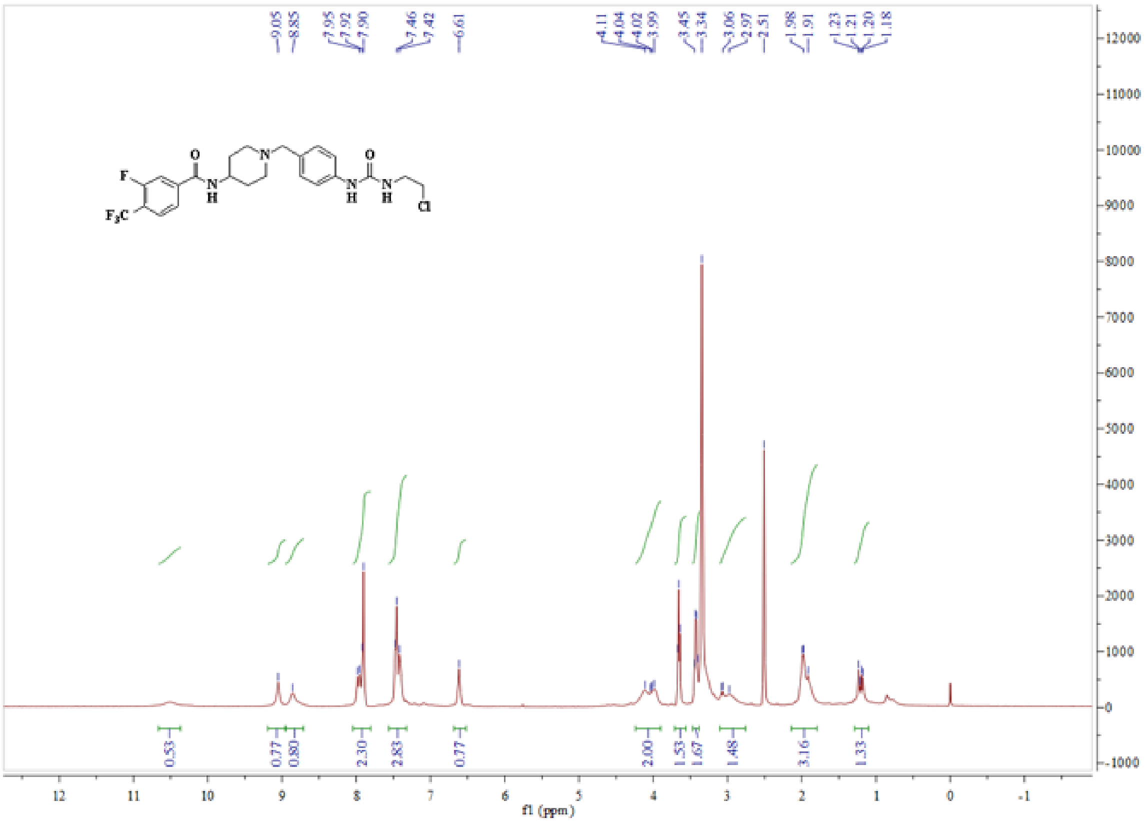

Figure S8. ^{1}H-NMR of compound **7i** in DMSO-d_6.

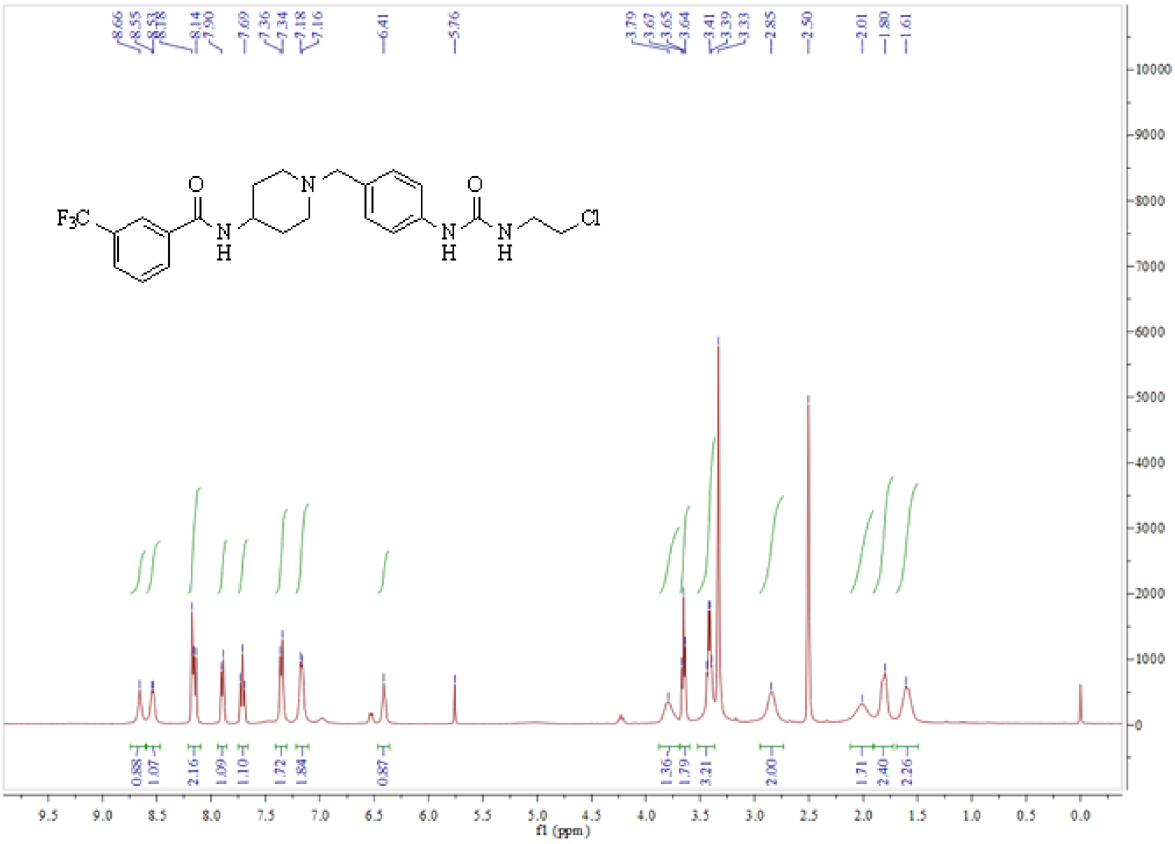

Figure S9. ^{1}H-NMR of compound **7j** in $CDCl_3$.

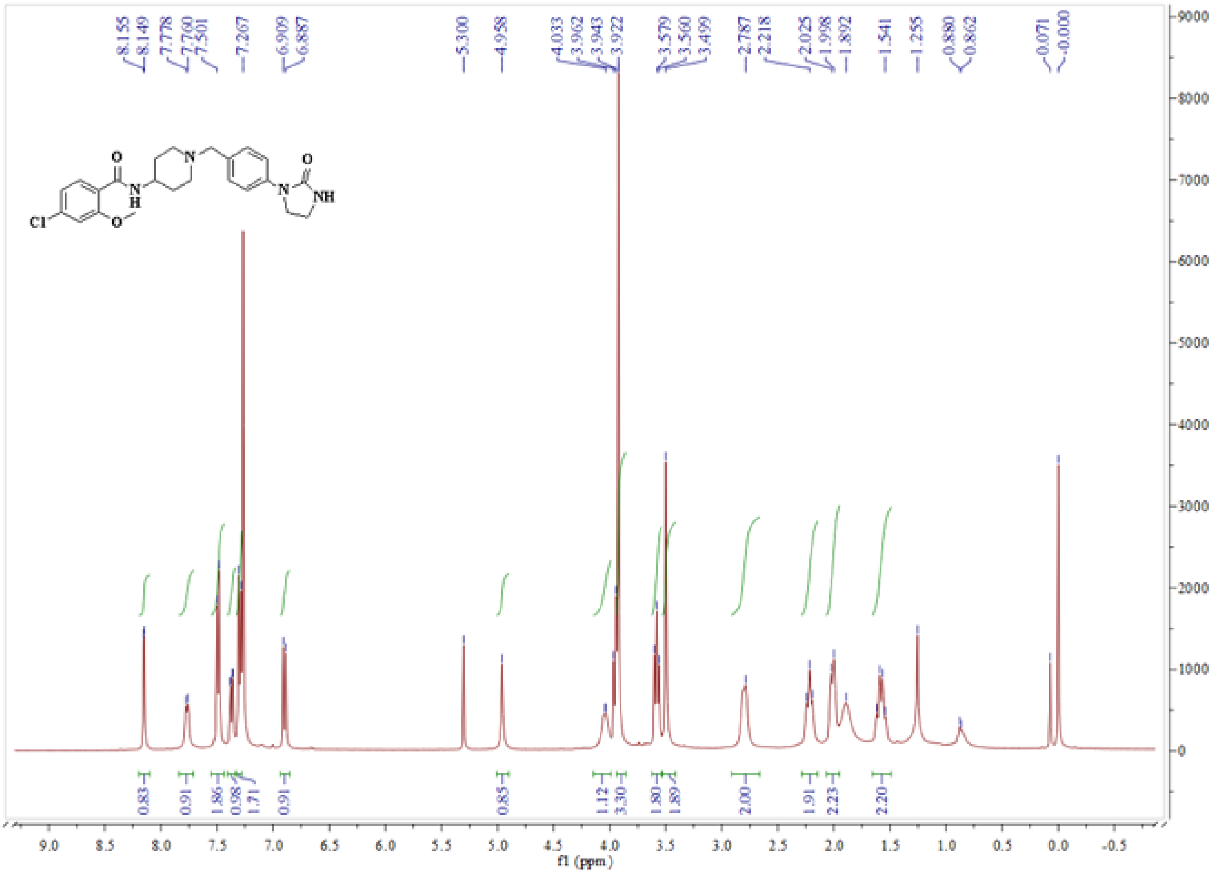

Figure S10. ^{1}H-NMR of compound **7k** in $CDCl_3$.

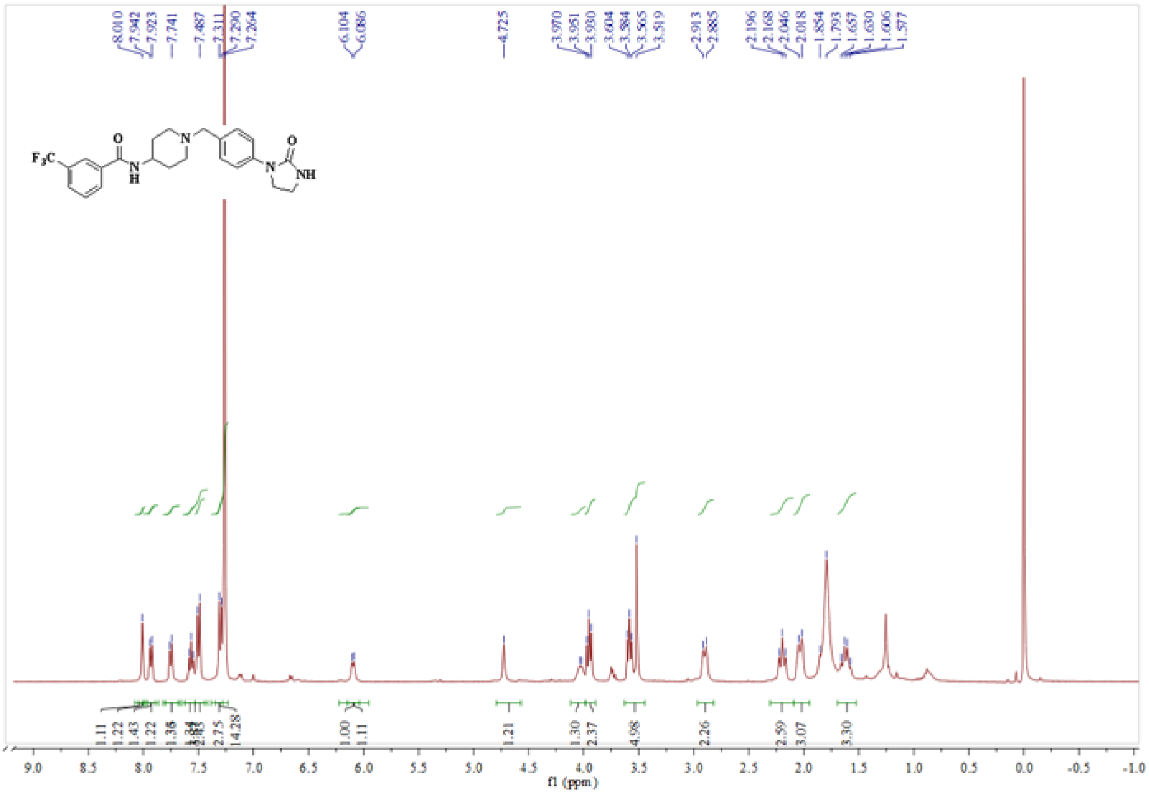

Figure S11. ^{1}H-NMR of compound **7l** in DMSO-d_6.

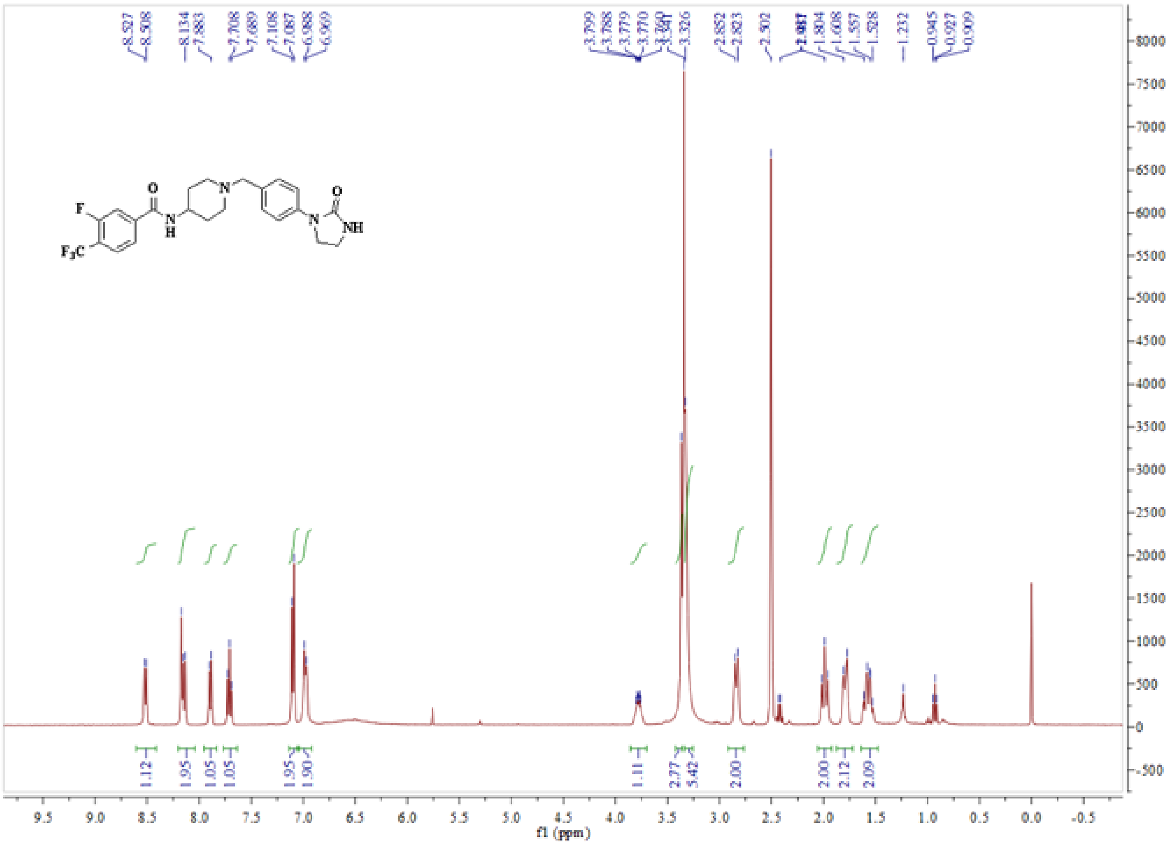

Figure S12. ^{1}H-NMR of compound **8a** in $CDCl_3$.

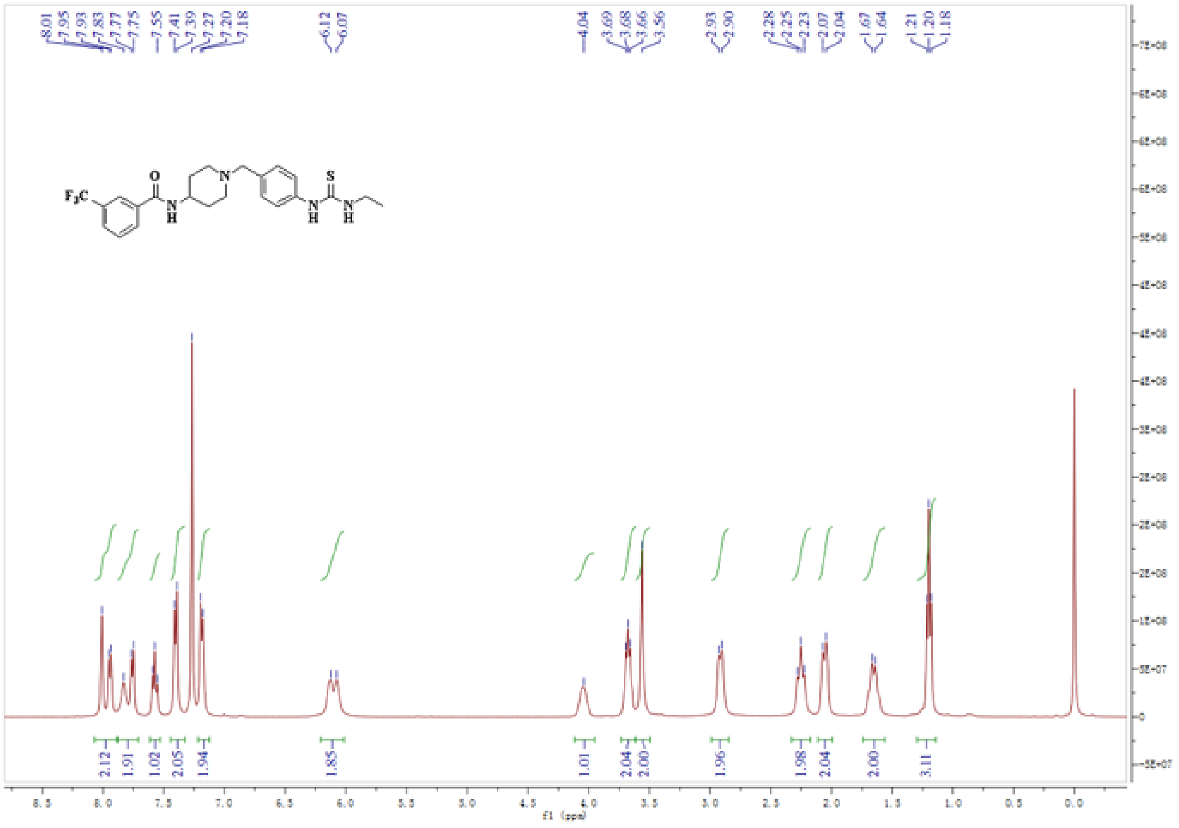

Figure S13. ^{1}H-NMR of compound **8b** in $CDCl_3$.

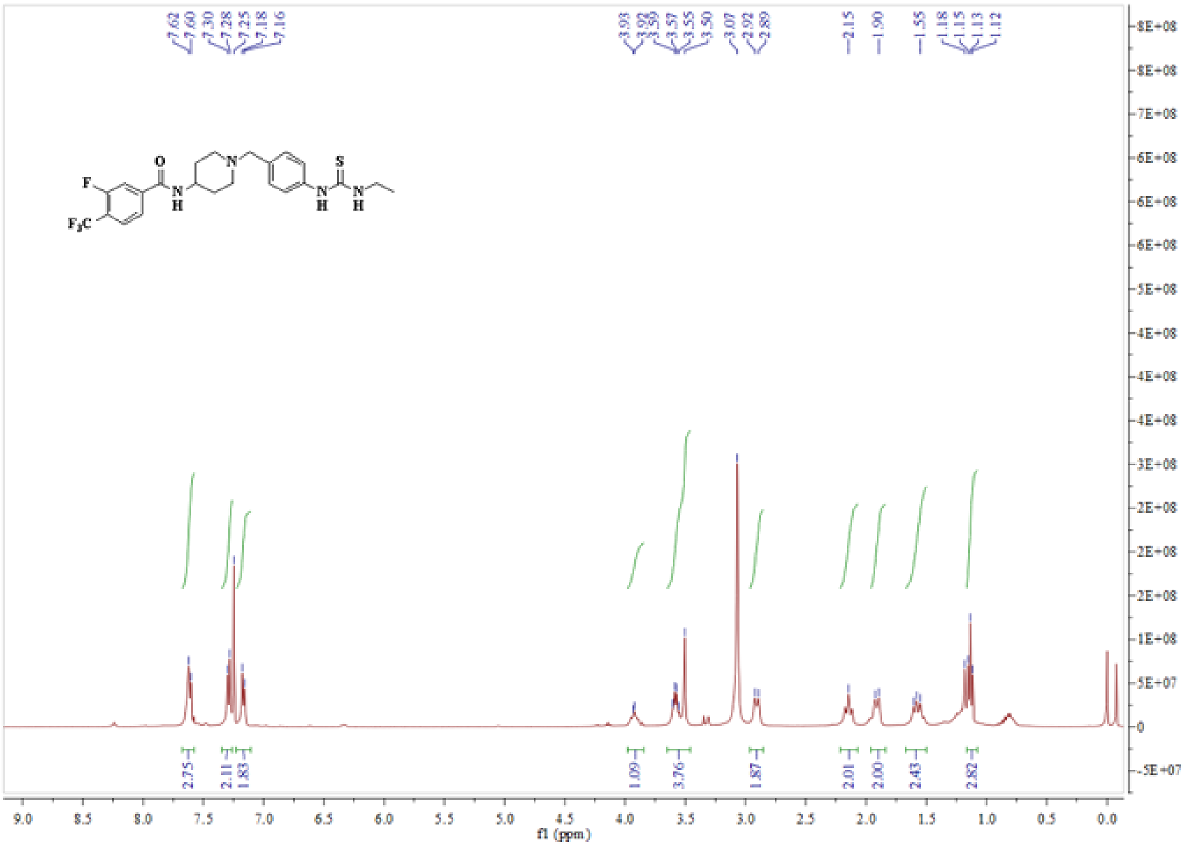

Figure S14. ^{1}H-NMR of compound **8c** in DMSO-d_6.

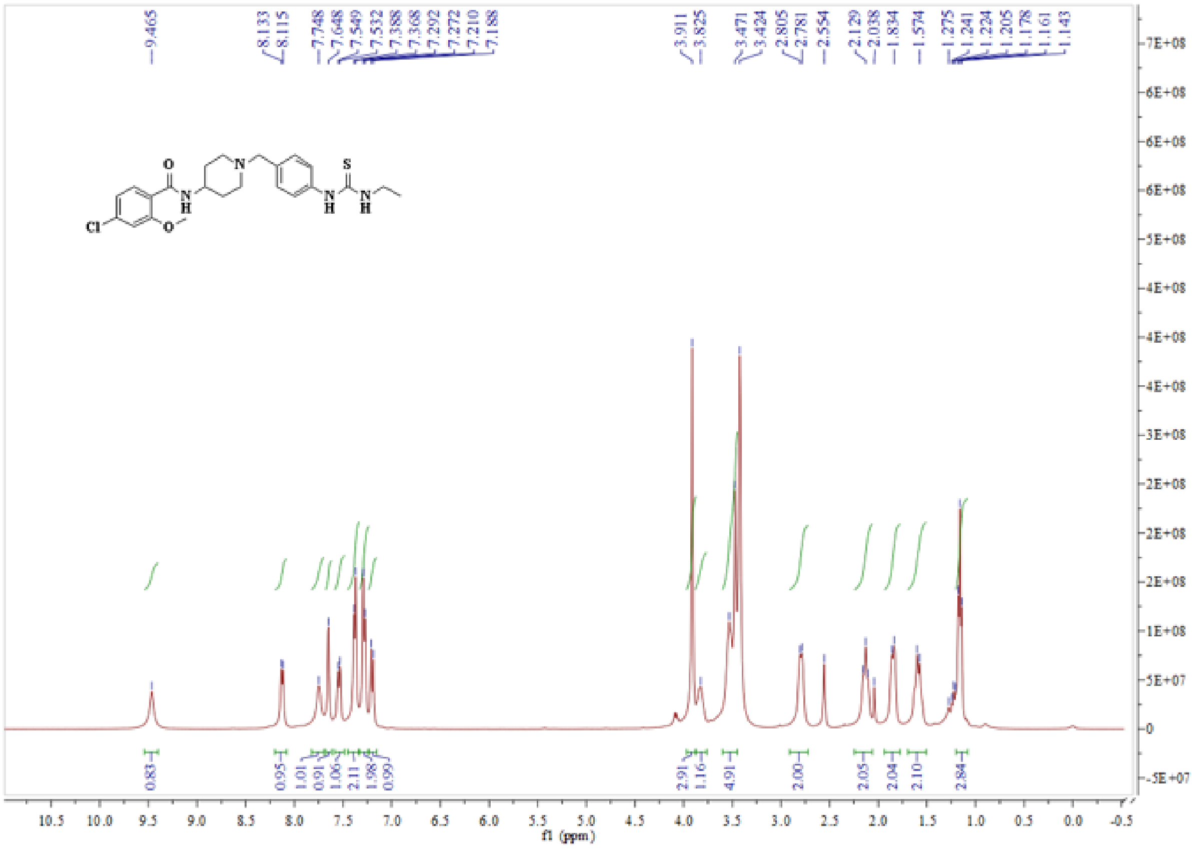

Figure S15. ^{1}H-NMR of compound **14a** in $CDCl_3$.

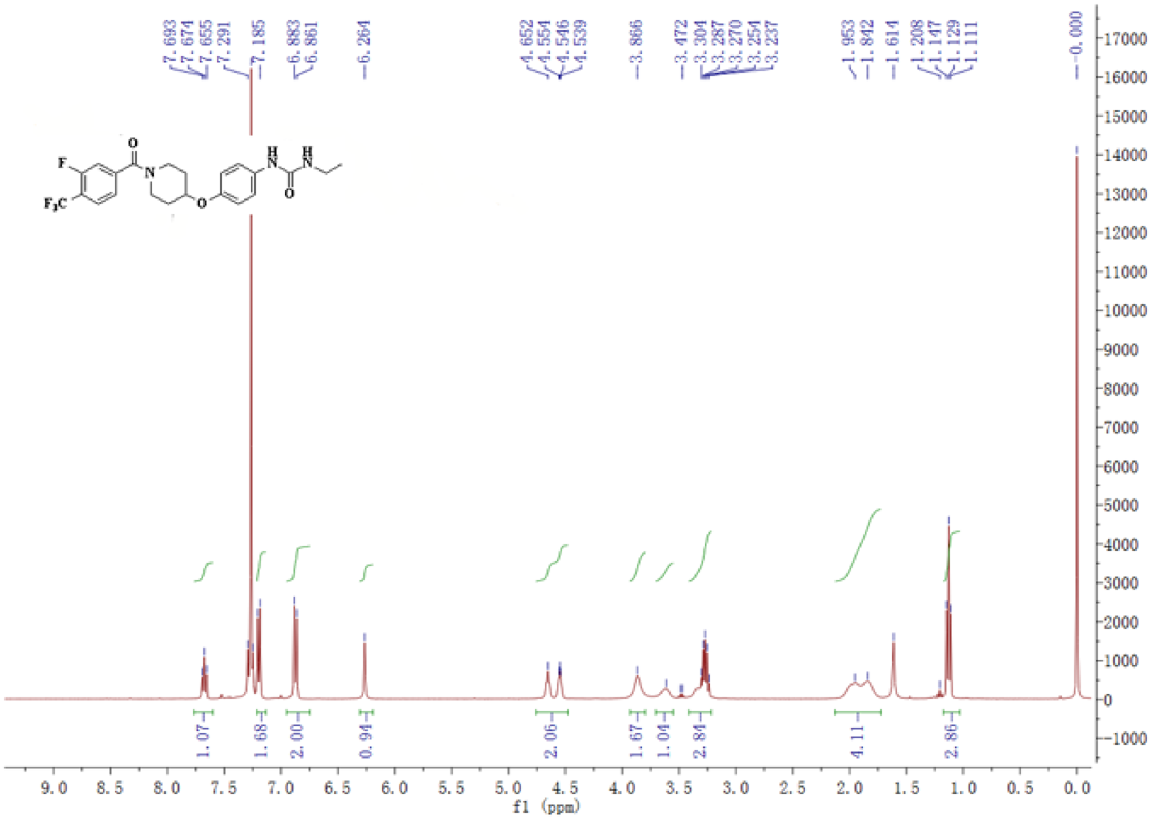

Figure S16. ^{1}H-NMR of compound **14b** in $CDCl_3$.

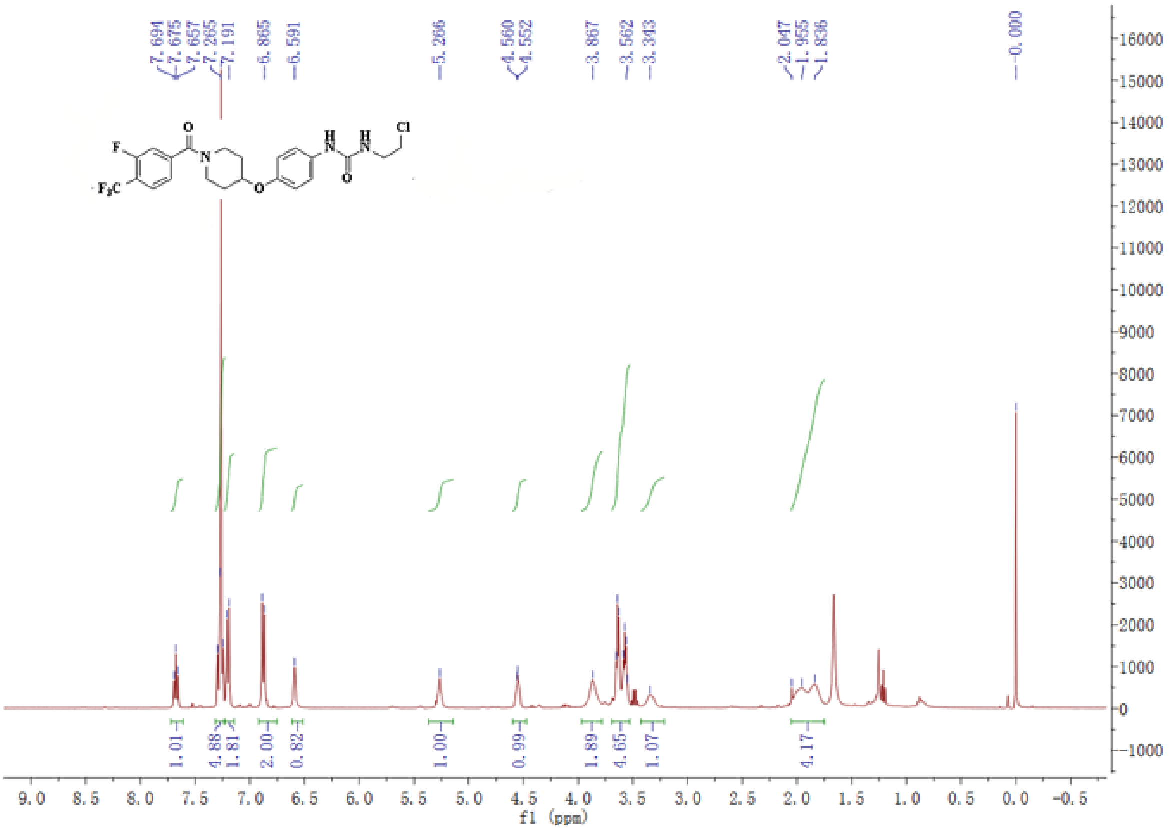

Figure S17. ^{1}H-NMR of compound **14c** in $CDCl_3$.

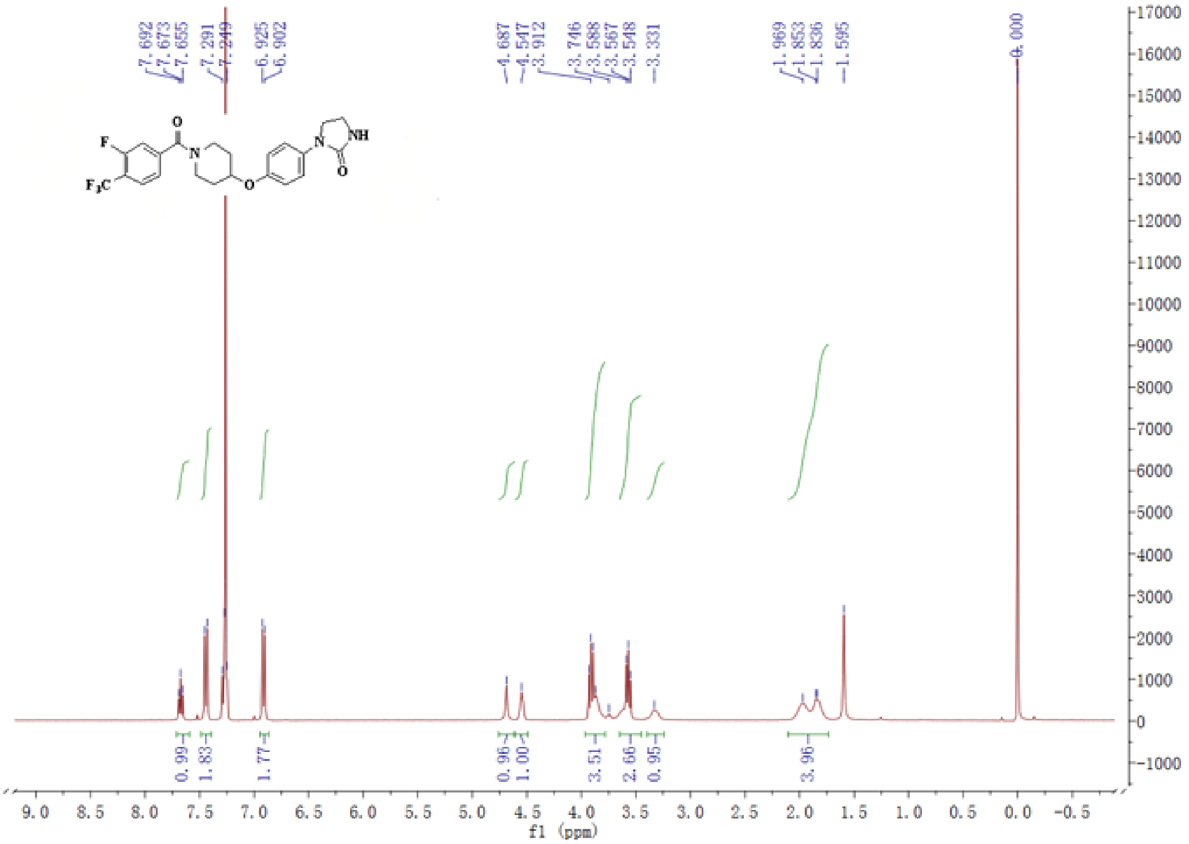

Figure S18. ^{1}H-NMR of compound **14d** in DMSO-d_6.

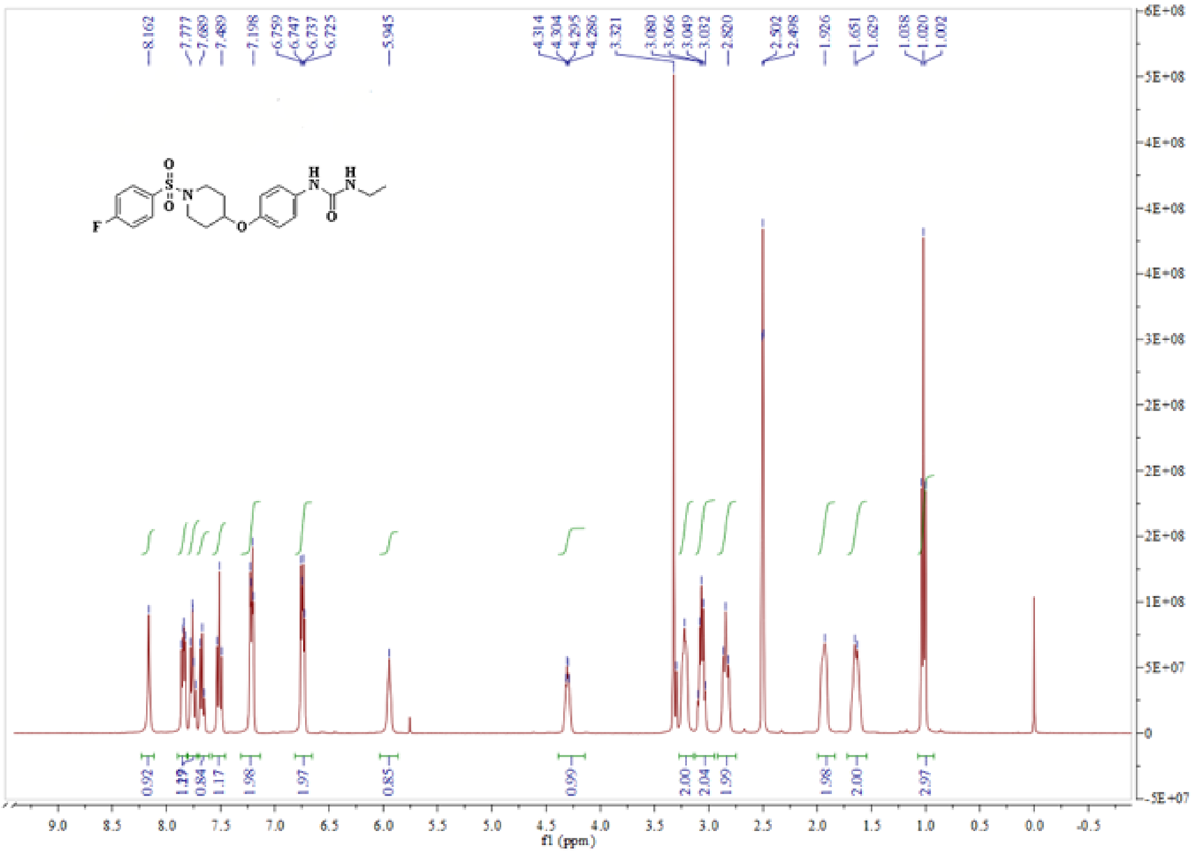

Figure S19. ^{1}H-NMR of compound 17**b** in DMSO-d_6.

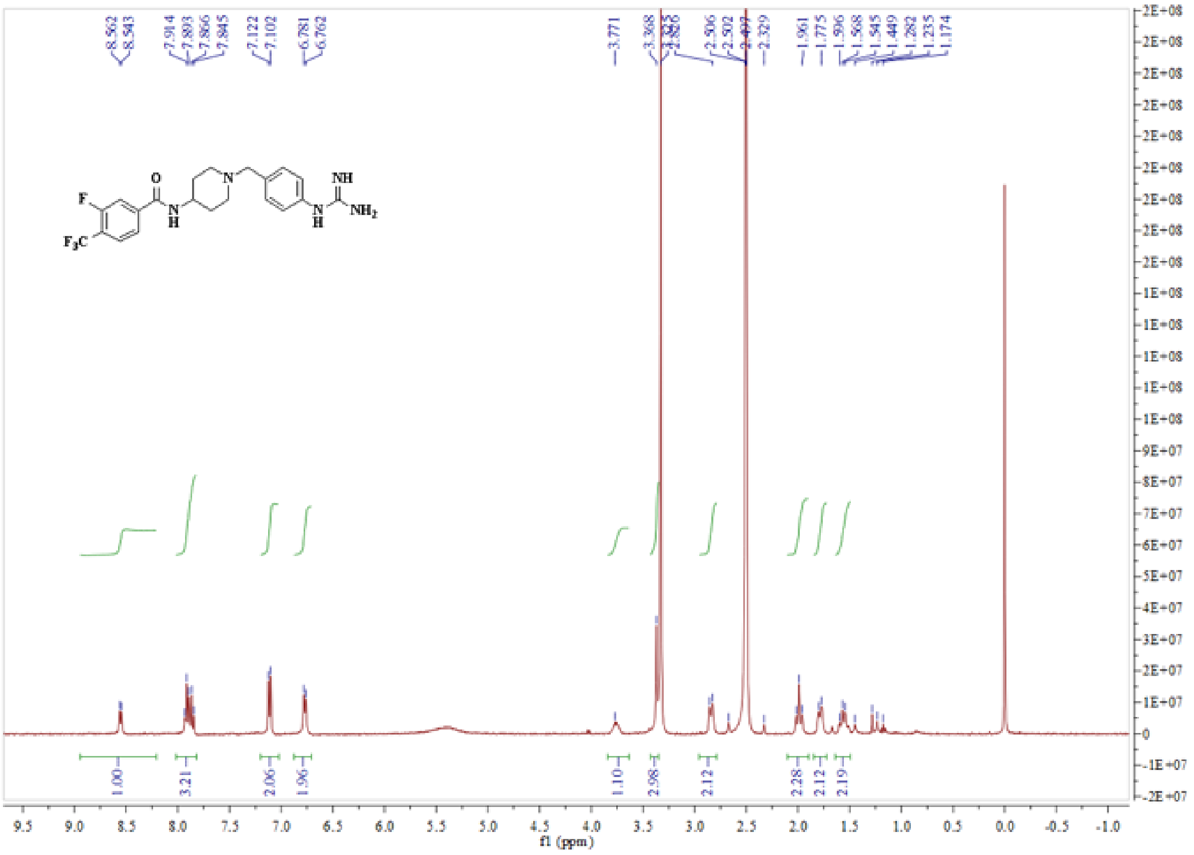

Figure S20. ^{1}H-NMR of compound 17**d** in $CDCl_3$.

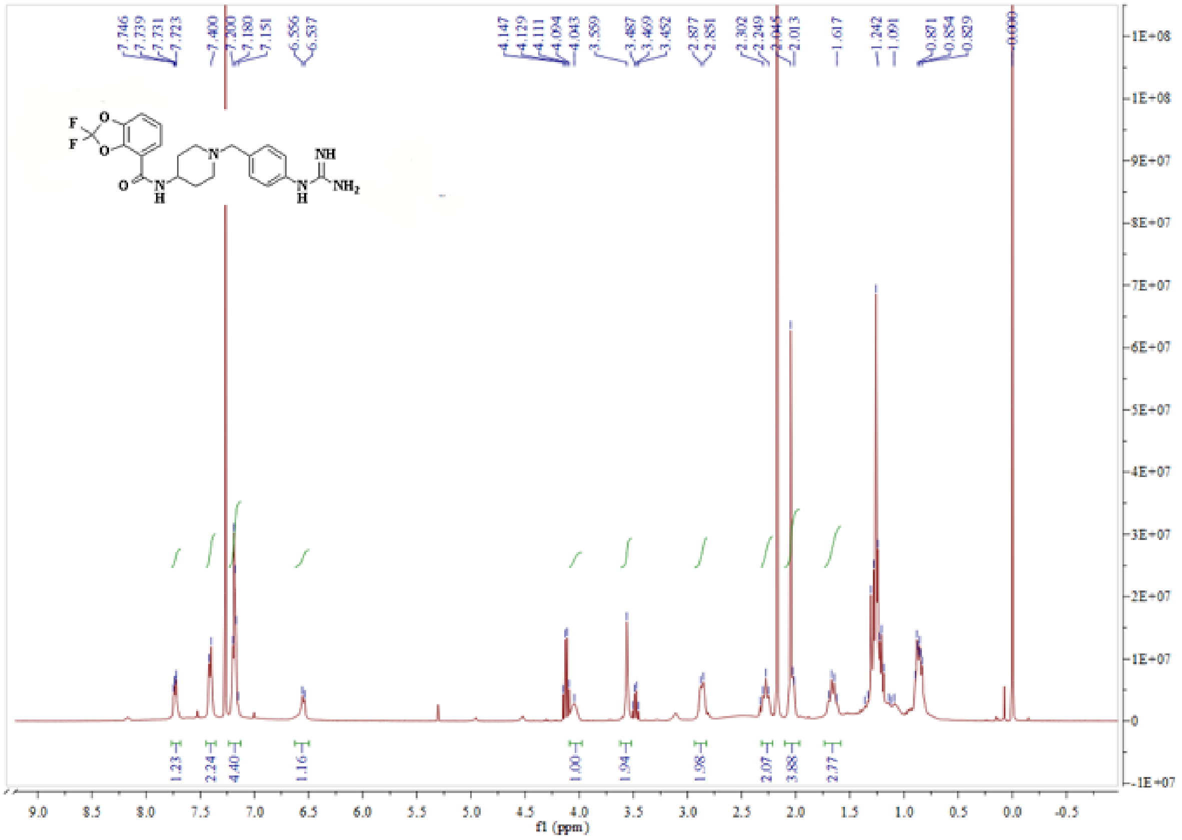

Figure S21. ^{1}H-NMR of compound 1**7f** in CD_3OD.

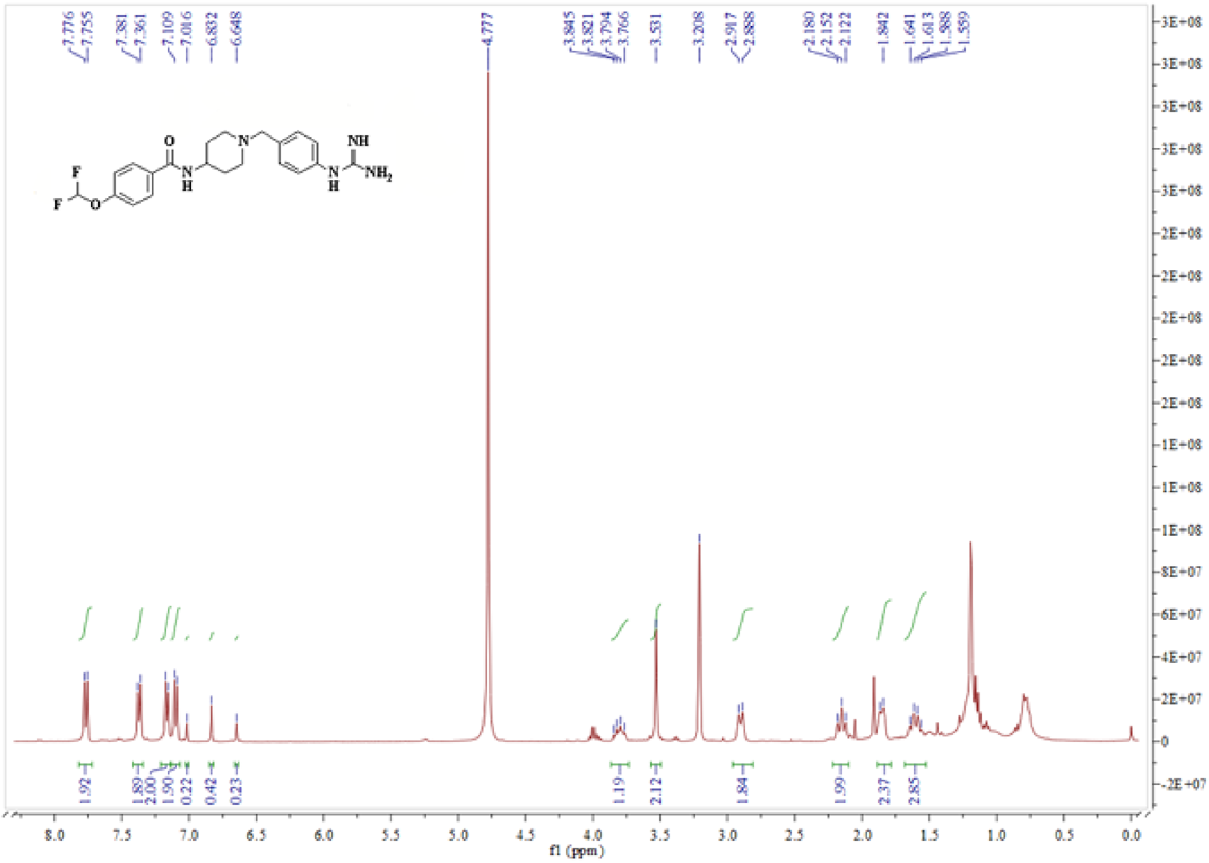

Figure S22. ^{1}H-NMR of compound 1**7g** in $CDCl_3$.

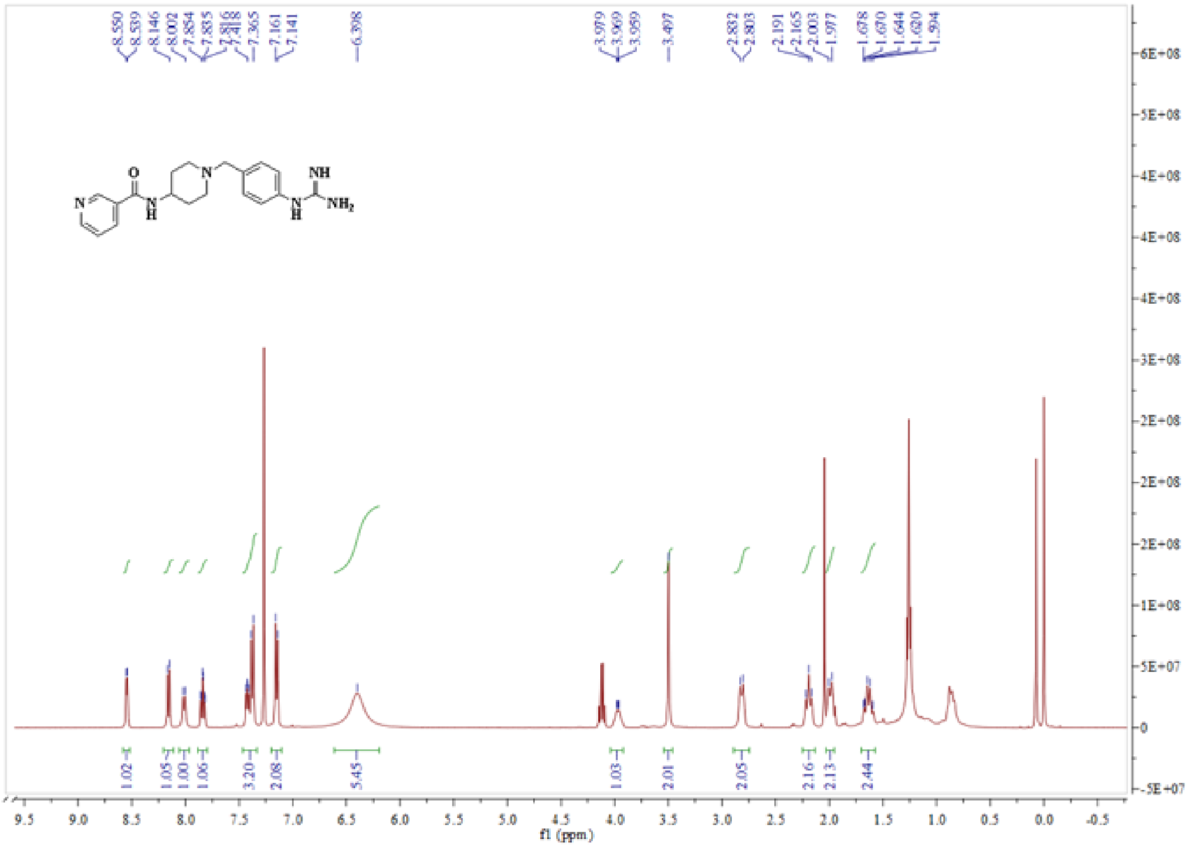

Figure S23. ^{1}H-NMR of compound 17**h** in $CDCl_3$.

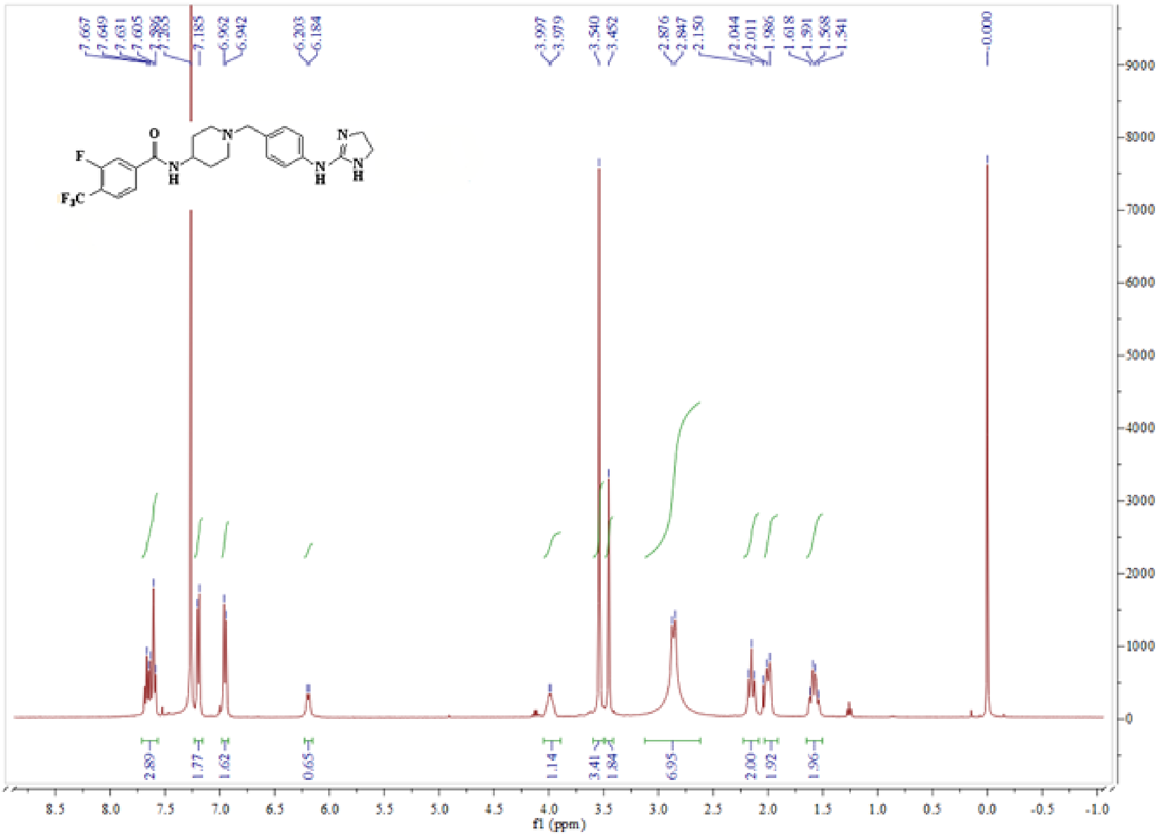

Figure S24. ^{1}H-NMR of compound 17**i** in $CDCl_3$.

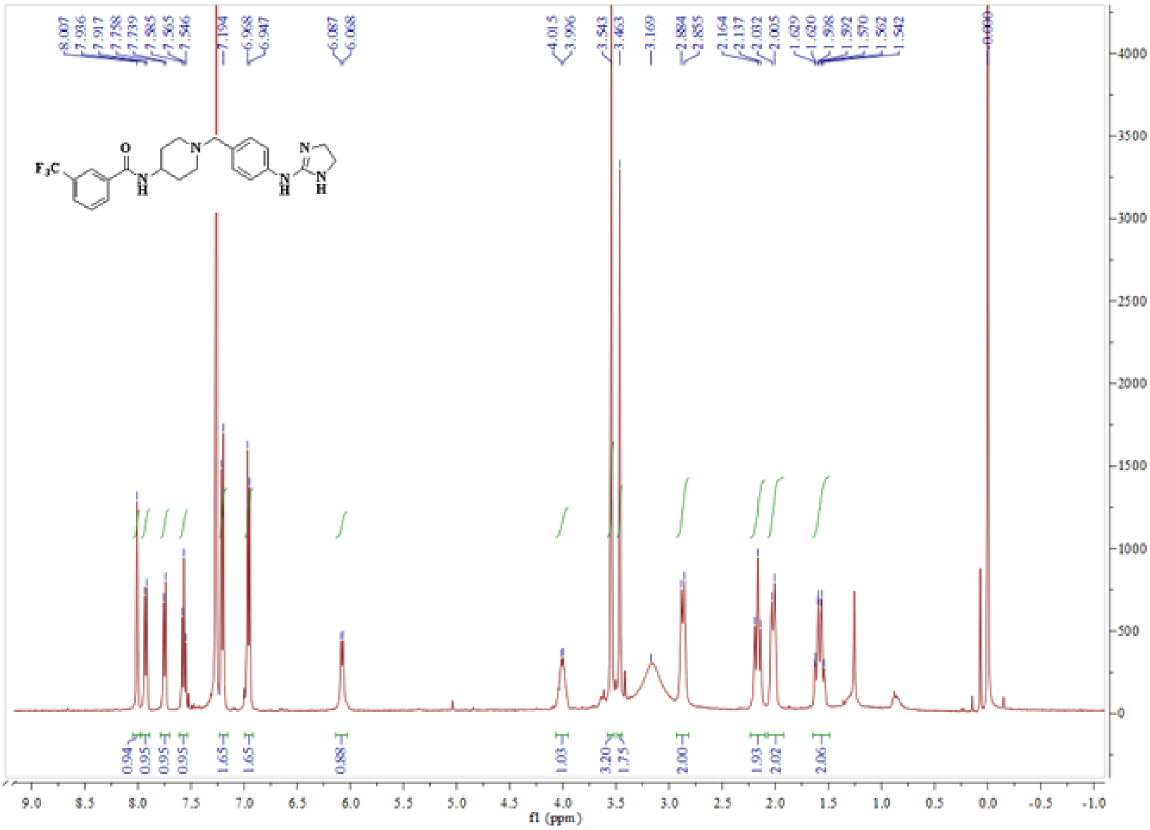

2. The ^{13}C-NMR and HPLC spectra of *N*-(1-(4-(3-(2-Chloroethyl)ureido)benzyl)piperidin-4-yl)-3-(trifluoromethyl) benzamide (**7i**).

Figure S25. The C^{13} spectrum of **7i**.

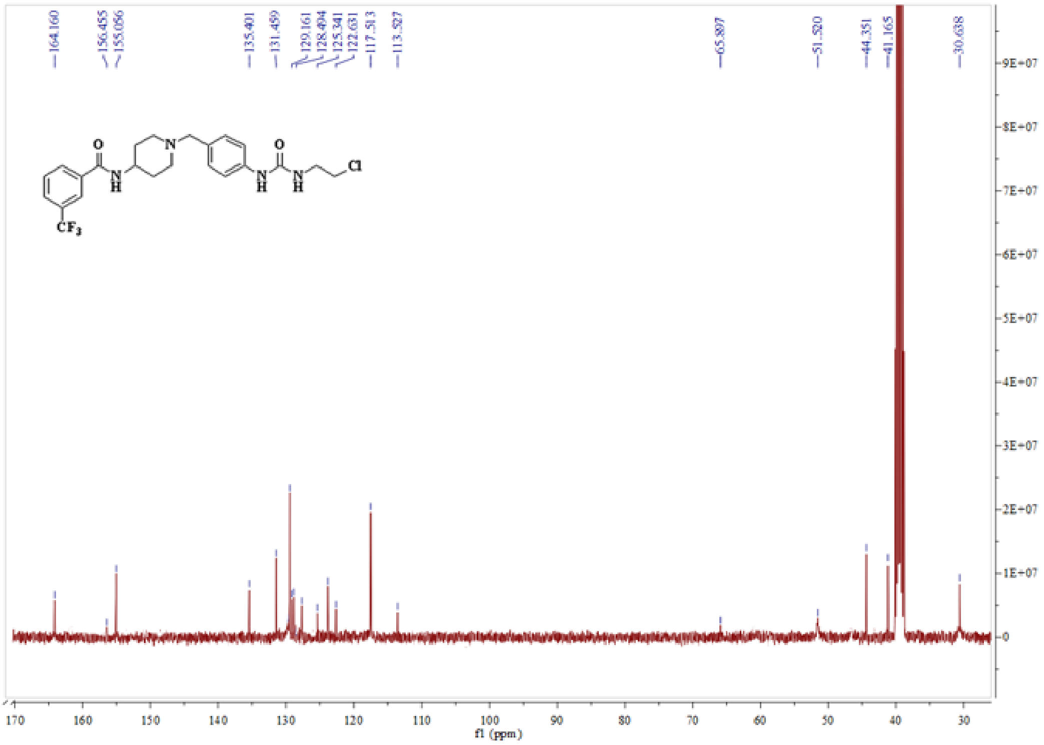

^{13}C-NMR (DMSO-d_6) δ: 30.6, 41.1, 44.3, 51.6, 65.9, 113.5, 117.4, 122.6, 123.7, 125.3, 127.6, 128.8, 129.2, 129.5, 131.5, 135.4, 155.1, 156.5, 164.2.

Figure S26. The HPLC spectrum of **7i**.

Empower™ 3 SOFTWARE

单个结果向导

样品信息

样品名称:	110-3	采集者:	System
样品类型:	未知	样品组名称:	
样品瓶:	43	采集方法组:	lxl
进样次数:	1	处理方法:	11003
进样体积:	10.00 ul	通道名称:	246.1 纳米
运行时间:	60.0 Minutes	处理通道说明:	PDA 246.1 纳米
采集时间:	2013/1/28 13:11:07 CST		
处理时间:	2013/1/28 14:15:06 CST		

自动缩放色谱图

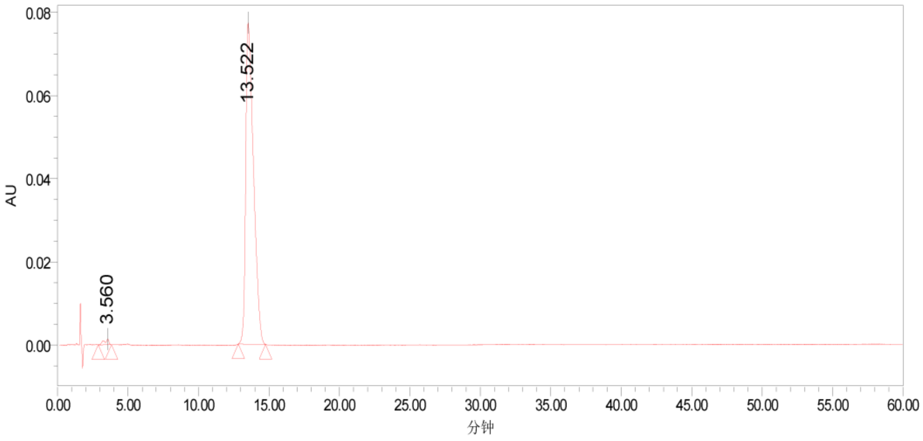

峰结果

	名称	保留时间 (分钟)	面积 (微伏*秒)	高度 (微伏)	% 面积
1		3.560	31940	1374	1.07
2		13.522	2955239	77339	98.93

报告用户: System　　　　项目名称: 梁晓琳
报告方法: 单个结果向导　　　　打印日期:
报告方法 ID: 241(2410　　　　2013/1/28
